# Linear and Angular Heteroannulated Pyridines Tethered 6-Hydroxy-4,7-Dimethoxybenzofuran: Synthesis and Antimicrobial Activity

**DOI:** 10.3390/molecules29184496

**Published:** 2024-09-22

**Authors:** Najla A. Alshaye, Al-Shimaa Badran, Magdy A. Ibrahim

**Affiliations:** 1 Department of Chemistry, College of Science, Princess Nourah Bint Abdulrahman University, P.O. Box 84428, Riyadh 11671, Saudi Arabia; naalshaye@pnu.edu.sa; 2 Department of Chemistry, Faculty of Education, Ain Shams University, Roxy, Cairo 11711, Egypt; elshimaabadran@edu.asu.edu.eg

**Keywords:** benzofuran, fused pyridines, 1,8-naphthyridine, cyclocondensation, nucleophilic reagents

## Abstract

2-Chloropyridine-3-carbonitrile derivative **1** was utilized as a key precursor to build a series of linear and angular annulated pyridines linked to a 6-hydroxy-4,7-dimethoxybenzofuran moiety. Reaction of substrate **1** with various hydrazines afforded pyrazolo[3,4-*b*]pyridines. Treatment of substrate **1** with 1,3-*N*,*N*-binucleophiles including 3-amino-1,2,4-triazole, 5-amino-1*H*-tetrazole, 3-amino-6-methyl-1,2,4-triazin-5(4*H*)-one and 2-aminobenzimidazole produced the novel angular pyrido[3,2-*e*][1,2,4]triazolo[4,3-*a*]pyrimidine, pyrido[3,2-*e*][1,2,4]tetrazolo[1,5-*a*]pyrimidine, pyrido[3′,2′:5,6] pyrimido[2,1-*c*][1,2,4]triazine and benzo[4,5]imidazo[1,2-*a*]pyrido[3,2-*e*]pyrimidine, respectively. Reaction of substrate **1** with 1,3-*C*,*N*-binucleophiles including cyanoacetamides and 1*H*-benzimidazol-2-ylacetonitrile furnished 1,8-naphthyridines and benzoimidazonaphthyridine. Moreover, reacting substrate **1** with 5-aminopyrazoles gave pyrazolo[3,4-*b*][1,8]naphthyridines. Finally, reaction of compound **1** with 6-aminouracils as cyclic enamines yielded pyrimido[4,5-*b*][1,8]naphthyridines. Some of the synthesized products showed noteworthy antimicrobial efficiency against all types of microbial strains. Structures of the produced compounds were established using analytical and spectroscopic tools.

## 1. Introduction

Benzofuran scaffolds, found in numerous natural products and medications, are of great therapeutic value [1,2,3]. Some drugs containing benzofurans with potential biological activities are approved by the USFDA or EMA [4]. Benzofurans constitute a valuable class in the field of drug discovery and development due to their interesting biological characteristics [5,6]. Noteworthy, substituted benzofurans exhibit significant efficiency against various tumors and cancer cell lines [7,8,9]. Also, benzofurans tethered different heterocyclic compounds displayed significant antimicrobial efficiency against a diversity of microbial strains [9,10,11,12]. Benzofuran derivatives also possess diverse biological properties, such as antioxidant, anti-inflammatory, antipyretic neuroprotective, analgesic as well treatment potential for Alzheimer’s diseases [13,14,15,16,17]. Theoretical studies and physical applications on benzofuran derivatives including HOMO-LUMO energy, MEP map, Mulliken atomic charges, dipole moments, solvatochromic, NBO and NLO, photophysical, photochemical and optoelectronic properties were also investigated [18,19,20,21,22]. A variety of synthetic strategies were developed to prepare heterocyclic compounds including benzofuran skeletons [23,24,25,26]. However, 6-substituted khellin represents an excellent building block to synthesize benzofuran-tethered heterocyclic systems due to the availability of electron-deficient γ-pyrone moiety [27,28,29,30,31]. On the other hand, a diversity of pyridines and their annulated heterocycles are widely synthesized using variable synthetic methodology [32,33,34,35]. Pyridine-based heterocycles exhibited promising pharmacological characteristics including antiviral, antiproliferative anticancer, antimicrobial, antimycobacterial, antifungal, anti-diabetic and anti-Alzheimer as well as inhibitors for acetylcholinesterase and butyrylcholinesterase [36,37,38,39,40,41,42]. Given the chemical and biological importance of benzopyrans and pyridines scaffolds, the current work aims to synthesize some new linear and angular annulated pyridines tethered to a 6-hydroxy-4,7-dimethoxybenzofuran moiety in one molecular frame utilizing *o*-choropyridinecarbonitrile **1** [43] as a building block, and to explore the biological activities of the prepared compounds.

## 2. Results and Discussion

### 2.1. Characterization of the Synthesized Compounds

It is known that compounds containing neighboring cyano and chloro functions are active building blocks for constructing nitrogen heterocyclic compounds [44,45]. Thus, chloropyridinecarbonitrile derivative **1** serves as an effective precursor for the synthesis of a variety of fused pyridines connected to a 6-hydroxy-4,7-dimethoxybenzofuranylcarbonyl moiety.

**Scheme 1:**
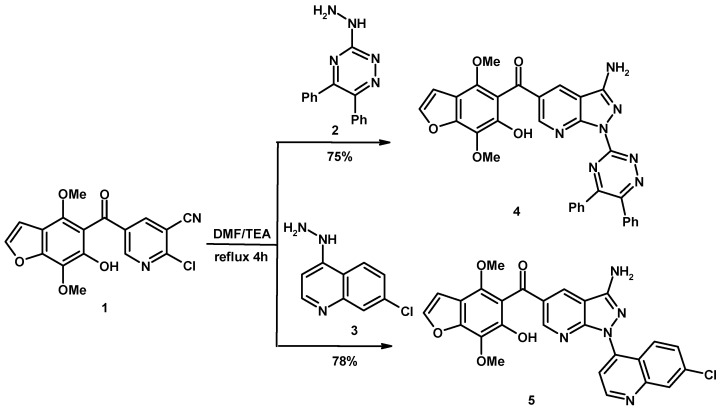
Formation of pyrazolo[3,4-*b*]pyridines **4** and **5**.

Reaction of substrate **1** with 3-hydrazino-5,6-diphenyl-1,2,4-triazine (**2**) [46] and 7-chloro-4-hydrazinoquinoline (**3**) [47], in refluxing DMF/TEA, afforded triazinyl/ quinolinylpyrazolo[3,4-*b*]pyridines **4** and **5**, respectively (Scheme 1). Compounds **4** and **5** are formed via the nucleophilic addition of NH_2_ group to the nitrile function in substrate **1**, followed by pyrazole ring closure and elimination of an HCl molecule. The mass spectra of compounds **4** and **5** confirmed their molecular formulae C_32_H_23_N_7_O_5_ and C_26_H_18_ClN_5_O_5_ showing their parent ion peaks at *m/z* 585 and 515, respectively. The C≡N function, detected at *ṽ* 2227 cm^−1^ in the spectrum of compound **1**, disappeared in the IR spectra of products **4** and **5**. The IR spectrum of products **4** and **5** presented distinctive absorption bands due to amino groups at *ṽ* 3368, 3293 and 3354, 3271 cm^−1^, respectively. Also, characteristic absorption bands corresponding to C=O and C=N were recorded at *ṽ* 1652/1658 and 1610/1612 cm^−1^. Further, the NH_2_ groups were detected in the ^1^H-NMR spectra of compounds **4** and **5** at δ 9.31 and 9.42 ppm, respectively, while the OH protons were seen at δ 12.29 and 12.52 ppm. In addition, two characteristic doublets attributable to H-3_furan_ and H-2_furan_ were seen in the ^1^H NMR spectra of compounds **4** and **5** at δ 7.08/7.13 and 7.85/7.86 ppm, respectively.

**Scheme 2:**
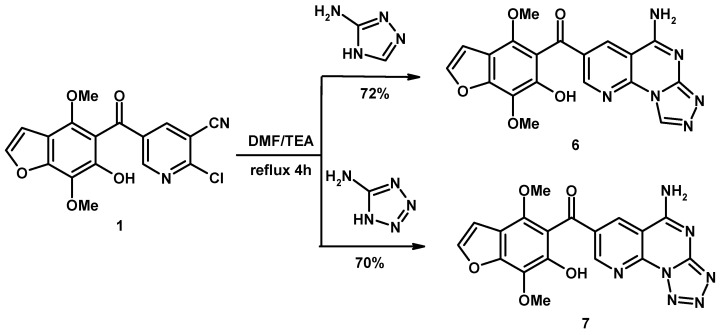
Formation of pyridotriazolopyrimidine **6** and pyridotetrazolopyrimidine **7**.

Likewise, compound **1** was permitted to react with some 1,3-*N*,*N*-binucleophiles. Thus, the novel angular pyrido[3,2-*e*][1,2,4]triazolo[4,3-*a*]pyrimidine **6** and pyrido[3,2-*e*][1,2,4]tetrazolo[1,5-*a*]pyrimidine **7** were synthesized from reacting substrate **1** with 3-amino-1,2,4-triazole and 5-amino-1*H*-tetrazole, respectively (Scheme 2). The mass spectra of the compounds **6** and **7** displayed their molecular ion peaks at *m/z* 406 and 407, coinciding with the proposed molecular formulae C_19_H_14_N_6_O_5_ and C_18_H_13_N_7_O_5_, respectively. Their IR spectra showed distinctive absorption bands at *ṽ* 3348, 3265/3369, 3287 (NH_2_) and 1649/1644 cm^−1^ (C=O). The ^1^H NMR spectra of compounds **6** and **7** revealed characteristic singlet signals due to H-4_pyridine_ and H-2_pyridine_ at δ 8.43/8.52 and 8.55/8.61, respectively. In addition, the amino protons were observed as D_2_O exchangeable signals at δ 9.50 and 9.26 ppm. The spectrum of compound **6** displayed definite singlet signal assignable to H-3_triazole_ at δ 8.97. The carbonyl carbon in compounds **6** and **7** were observed in the downfield region in the ^13^C NMR spectra at δ 192.3 and 192.4 ppm, respectively, also the spectrum of compound **6** showed distinctive singlet due to C-3_triazole_ at δ 137.3 ppm.

**Scheme 3:**
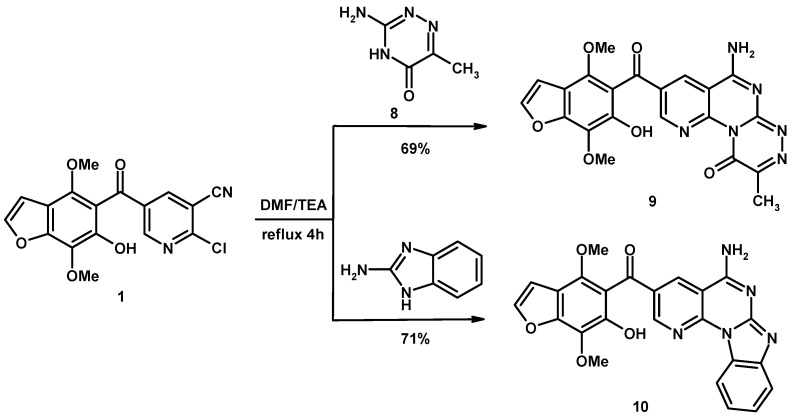
Formation of angular pyridopyrimidotriazine **9** and benzoimidazopyrido-pyrimidine **10**.

Similarly, treatment of substrate **3** with 3-amino-6-methyl-1,2,4-triazin-5(4*H*)-one (**8**) [48] and 2-aminobenzimidazole, in boiling DMF/TEA, yielded the novel angular annulated pyrido[3′,2′:5,6]pyrimido[2,1-*c*][1,2,4]triazine **9** and benzo[4,5]imidazo[1,2-*a*] pyrido[3,2-*e*]pyrimidine **10**, respectively (Scheme 3). The IR spectra of compounds **9** and **10** showed distinctive absorption bands at *ṽ* 3372,3296/3383,3268 (NH_2_) and 1654/1648 cm^−1^ (C=O). Also, the spectrum of compound **9** presented distinguish absorption band due to C=O_triazine_ at *ṽ* 1692 cm^−1^. The ^1^H NMR spectra of compounds **9** and **10** presented D_2_O exchangeable signals due to amino protons at δ 9.32 and 9.52 ppm, respectively. The spectrum of compound **9** displayed an upfield signal at δ 2.18, corresponding to CH_3 triazine_. The ^13^C NMR spectrum of compound **9** showed two specific signals attributed to CH_3 triazine_ and C=O_triazine_ at δ 17.3 and 166.2 ppm. The mass spectra of compounds **9** and **10** showed their molecular ion peaks at *m/z* 448 and 455, respectively, which coincided well with their proposed molecular formulae C_21_H_16_N_6_O_6_ and C_24_H_17_N_5_O_5_, respectively.

**Scheme 4:**
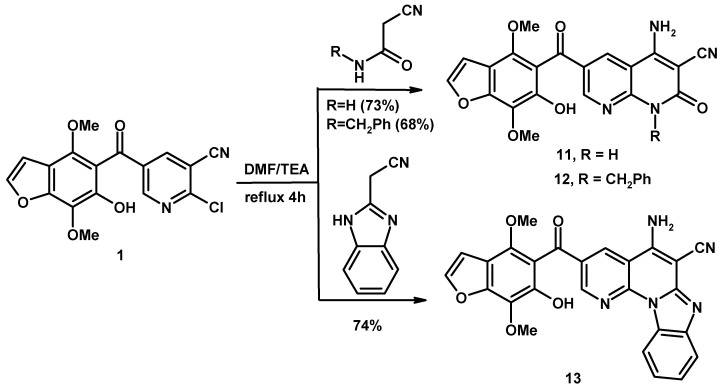
Formation of naphthyridines **11**, **12** and benzoimidazonaphthyridine **13**.

Next, compound **1** was permitted to react with some of 1,3-*C*,*N*-binucleophiles. Hence, reaction of compound **1** with cyanoacetamide, *N*-benzyl-2-cyanoacetamide and 1*H*-benzimidazol-2-ylacetonitrile, in boiling DMF/TEA, furnished 1,8-naphthyridine-3-carbonitriles **11**, **12** and benzo[4,5]imidazo[1,2-*a*][1,8] naphthyridine-6-carbonitrile **13**, respectively (Scheme 4). The IR spectra of compounds **11–13** showed characteristic absorption bands attributed to C≡N at *ṽ* 2224, 2221 and 2226 cm^−1^, respectively. The spectra of compounds **11** and **12** showed characteristic absorption bands due to C=O_pyridine_ at 1681 and 1686 cm^−1^. The ^1^H NMR spectra of compounds **11–13** presented the NH_2_ protons as exchangeable signals at δ 9.32, 9.28 and 9.41 ppm, respectively. The NH proton in compound **11** was seen at δ 11.04 ppm. Also, the CH_2_ protons in compound **12** were recorded at δ 3.08 ppm. The ^13^C NMR spectra of compounds **11** and **12** showed characteristic signals attributed to C≡N, C=O_naphthyridine_ and C=O_ketone_ at δ 116.3/116.6, 169.5/169.1 and 193.2/194.1 ppm, respectively. The spectrum of compound **12** displayed the methylene carbon as definite signal at δ 29.4. The mass spectra of compounds **11–13** exhibited their parent ion peaks at *m/z* 406, 496 and 479 that agree well with the suggested molecular formulae C_20_H_14_N_4_O_6_ (406.35), C_27_H_20_N_4_O_6_ (496.47) and C_26_H_17_N_5_O_5_ (479.44), respectively.

**Scheme 5:**
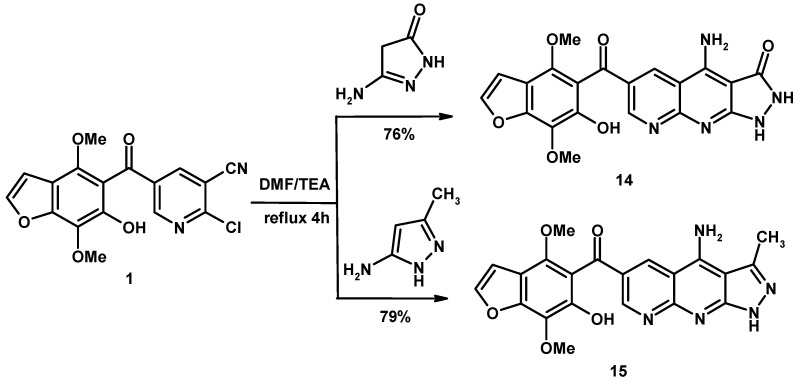
Formation of pyrazolo[3,4-*b*][1,8]naphthyridines **14** and **15**.

Moreover, the reaction of substrate **1** with 5-amino-2,4-dihydro-3*H*-pyrazol-3-one and 5-amino-3-methyl-1*H*-pyrazole, in boiling DMF/TEA, gave linear annulated pyrazolo[3,4-*b*][1,8]naphthyridines **14** and **15**, respectively (Scheme 5). The IR spectrum of compound **14** showed typical absorption bands at *ṽ* 3376, 3338, 3296 (NH_2_, 2NH), 1667 (C=O_pyrazole_) and 1646 cm^−1^ (C=O). The ^1^H NMR spectrum of compound **15** revealed characteristic singlet signals at δ 2.42, 8.52 and 8.64 ppm attributed to CH_3 pyrazole_, H-4_pyridine_ and H-2_pyridine_, in addition to three D_2_O exchangeable signals at δ 9.32 (NH_2_), 10.36 (NH) and 12.41 ppm (OH). The mass spectra of compounds **14** and **15** revealed their molecular ion peaks at *m/z* 421 and 319 that match well with the proposed molecular formulae C_20_H_15_N_5_O_6_ (421.36) and C_21_H_17_N_5_O_5_ (419.39), respectively. The carbon of C=O_pyrazole_ was seen in the ^13^C NMR spectrum of compound **14** in the downfield region at δ 165.5 ppm, while the spectrum of compound **15** presented distinctive signal due to CH_3 pyrazole_ at the upfield region δ 18.6 ppm (CH_3_).

**Scheme 6:**
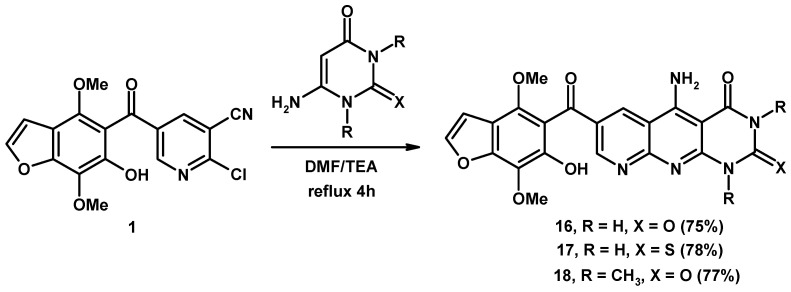
Formation of pyrimido[4,5-*b*][1,8]naphthyridines **16**–**18**.

Finally, compound **1** was permitted to react with some cyclic enamines namely 6-aminouracil, 6-aminothiouracil and 6-amino-1,3-dimethyluracil, in DMF containing TEA, giving pyrimido[4,5-*b*][1,8]naphthyridines **16**–**18**, respectively (Scheme 6). The mass spectra of compounds **16**–**18** presented their molecular ion peaks at *m/z* 449, 465 and 477 approving their suggested formula weights 449.37 (C_21_H_15_N_5_O_7_), 465.44 (C_21_H_15_N_5_O_6_S) and 477.43 (C_23_H_19_N_5_O_7_), respectively. The amino protons were observed in ^1^H NMR spectra of compounds **16**–**18** at δ 9.58, 9.34 and 9.37 ppm, respectively. Two characteristic signals attributable to 2NCH_3_ protons were seen in the ^1^H NMR spectrum of compound **18** at δ 3.06 and 3.17 ppm. Further, the ^13^C NMR spectra of compounds **16** and **18** showed characteristic signals at δ 165.1/165.5 (C2 as C=O_pyrimidine_) and 167.4/168.1 (C4 as C=O_pyrimidine_), while The spectrum of compound **17** displayed specific signals due to C4 as C=O_pyrimidine_ and C2 as C=S_pyrimidine_ at δ 168.9 and 186.3 ppm, respectively. Also, the ^13^C NMR spectrum of compound **18** showed two characteristic signals at δ 28.9 and 30.0 corresponding to 2NCH_3_ carbons.

### 2.2. Antimicrobial Estimation

The synthesized products were investigated for their antimicrobial assay, in vitro, against some Gram-positive bacteria (*S. aureus* and *B. subtilis*) and Gram-negative bacteria (*S. typhimurium* and *E. coli*), as well as yeast (*C. albicans*) and fungus (*A. fumigatus*).

To assess the antimicrobial efficacy of the synthesized products, the inhibitory zones, including the disc diameter (6 mm), were evaluated (Table 1) [49]. High inhibition action referred to zone diameter >2/3 zone diameter of control, while moderate activity means zone diameter ≤2/3 and >1/3 zone diameter of reference drug. Cycloheximide is the reference drug for fungus and yeast, while Chloramphencol for Gram-positive bacteria, and Cephalothin for Gram-negative bacteria.

**Chart 1:**
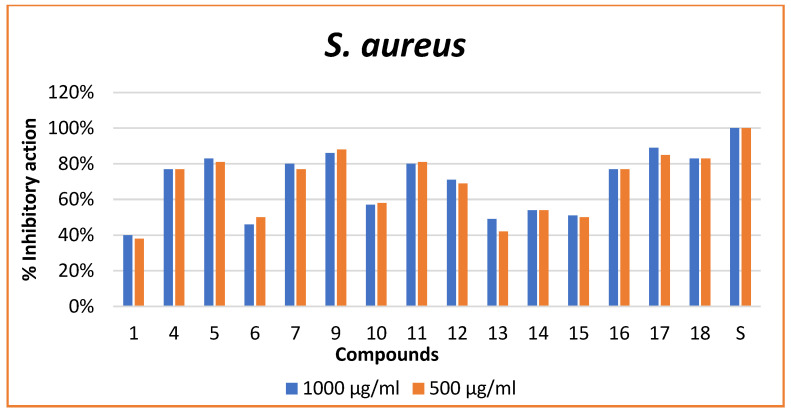
The antibacterial efficiency of synthesized compounds against *S. aureus*.

According to the results in Table 1 (Chart 1 and Charts S1–S5), all examined compounds had a strong inhibitory impact on the tested strains of fungus and yeast; this may due to the presence of the 6-hydroxy-4,7-dimethoxy-1-benzofuran moiety which exists in all products. Meanwhile, the inhibitory effect against the microbial strains varies according to the effect of the synthesized heterocyclic rings. Compounds **4** and **5** presented high efficiency against both types of Gram-positive and Gram-negative bacteria and this may be attributed to the presence of triazinyl/quinolinyl-pyrazolopyridine moieties linked to the benzofuranylcarbonyl fragment. Also, building angular heterocyclic systems, namely pyridotetrazolopyrimidine **7** and pyridopyrimidotriazine **9**, enhanced the inhibitory effects against all tested microorganisms. On the other hand, some linear heterocyclic systems such as 1,8-naphthyridines **11**, **12** and pyrimidonaphthyridines **16**–**18** showed high inhibition actions towards all tested bacterial strains.

As illustrated above, due to the existence of the principal scaffold, 6-hydroxy-4,7-dimethoxy-1-benzofuran moiety, all of the examined products demonstrated valuable inhibitory effects towards yeast and fungus. Furthermore, the inhibitory action towards bacterial strains was improved by the inclusion of additional heterocyclic systems, such as pyrazolopyridine, pyridotetrazolopyrimidine, pyridopyrimidotriazine, 1,8-naphthyridine and pyrimidonaphthyridine. As a result, some of the produced compounds may have excellent antimicrobial properties. 

## 3. Materials and Methods

### 3.1. General Information

General. Melting point determination was performed using a digital Stuart SMP3 device (Buchi, Flawil, Switzerland). The mass spectra were measured using Shimadzu (Tokyo, Japan) GC-2010 mass spectrometer (70 eV); in gas chromatography. The ^1^H NMR (400 MHz) and ^13^C NMR (100 MHz) spectra were measured with the Mercury-400BB apparatus (vnmr1, Rheinstetten, Germany) using DMSO-d_6_ as the solvent and TMS (δ) as the internal standard. Using KBr disks, an FTIR Nicolet (Green Bay, WI, USA) IS10 spectrophotometer (cm^−1^) was used to record the infrared spectra. 2-Chloro-5-[(6-hydroxy-4,7-dimethoxy-1-benzofuran-5-yl)carbonyl]pyridine-3-carbonitrile (**1**) was prepared according to literature [43].

### 3.2. Biological Method

On medium potato dextrose agar (PDA), which comprised an infusion of 200 g potatoes, 6 g dextrose, and 15 g agar, the antimicrobial activity test was conducted. Filter paper disks of uniform size (6 mm in diameter, with three disks for each chemical) were carefully placed on an inoculated agar surface after being impregnated with an equivalent volume (10 µL) of dissolved compounds at concentrations of 500 and 1000 mg/mL in dimethylformamide (DMF). Following 36 h of incubation at 27 °C for bacteria and 48 h at 24 °C for fungi. The average diameter of the bacterial and fungal inhibitory zones surrounding the disks, measured in millimeters at concentrations of 500 and 1000 mg/mL, was recorded for each investigated compound [49].

3-Amino-1-(5,6-diphenyl-1,2,4-triazin-3-yl)-5-[(6-hydroxy-4,7-dimethoxy-1-benzofuran-5-yl)carbonyl]-1*H*-pyrazolo[3,4-*b*]pyridne (**4**)

A mixture of compound **1** (0.72 g, 2 mmol) and 3-hydrazinyl-5,6-diphenyl-1,2,4-triazine (**2**) (0.58 g, 2 mmol), in DMF (10 mL) containing TEA (0.1 mL), was heated under reflux for 4 h. After cooling, the pale-yellow crystals deposited were filtered and recrystallized from AcOH, mp > 300 °C, yield (0.88 g, 75%). IR (KBr, cm^−1^): 3413 (OH), 3368, 3293 (NH_2_), 1652 (C=O), 1610 (C=N), 1581 (C=C). ^1^H NMR (400 MHz, DMSO-*d*_6_, δ): 3.86 (s, 3H, OCH_3_), 3.95 (s, 3H, OCH_3_), 7.08 (d, 1H, *J* = 2.0 Hz, H-3_furan_), 7.44–7.52 (m, 10H, Ar-H), 7.85 (d, 1H, *J* = 2.0 Hz, H-2_furan_), 8.42 (s, 1H, H-4_pyridine_), 8.48 (s, 1H, H-2_pyridine_), 9.31 (s, 2H, NH_2_ exchangeable with D_2_O), 12.29 (s, 1H, OH exchangeable with D_2_O). ^13^C NMR (75 MHz, DMSO-*d*_6_, δ): 58.7 (OMe), 59.8 (OMe), 102.3 (C3a′), 104.0 (C3′), 108.2 (C3a), 112.4 (C5′), 121.4 (Ar-C), 122.1 (Ar-C), 123.8 (C7′), 124.7 (Ar-C), 125.2 (Ar-C), 127.6 (Ar-C), 129.1 (C4′), 129.8 (Ar-C), 131.4 (Ar-C), 132.8 (C5), 135.7 (Ar-C), 138.2 (C-7a), 139.0 (C-5_triazine_), 139.9 (C-6_triazine_), 140.2 (C-3_triazine_), 143.2 (C-3), 144.5 (C-4), 147.2 (C-2′), 148.1 (C-6), 149.1 (C6′), 151.6 (C7a′), 189.7 (C=O_ketone_). Mass spectrum, *m/z* (*I*_r_ %): 585 (M^+^, 46), 555 (24), 452 (37), 353 (16), 324 (20), 220 (71), 178 (100), 159 (13), 133 (10), 117 (16), 93 (25), 77 (48), 64 (21). Anal. Calcd for C_32_H_23_N_7_O_5_ (585.57): C, 65.64; H, 3.96; N, 16.74%. Found: C, 65.37; H, 3.88; N, 16.64%.

3-Amino-1-(7-chloroquinolin-4-yl)-5-[(6-hydroxy-4,7-dimethoxy-1-benzofuran-5-yl)carbonyl]-1*H*-pyrazolo[3,4-*b*]pyridne (**5**)

A mixture of compound **1** (0.72 g, 2 mmol) and 7-chloro-4-hydrazinylquinoline (**3**) (0.38 g, 2 mmol), in DMF (10 mL) containing TEA (0.1 mL), was heated under reflux for 4 h. After cooling, the orange-yellow crystals so formed were filtered and recrystallized from AcOH, mp > 300 °C, yield (0.79 g, 78%). IR (KBr, cm^−1^): 3408 (OH), 3354, 3271 (NH_2_), 1658 (C=O), 1612 (C=N), 1588 (C=C). ^1^H NMR (400 MHz, DMSO-*d*_6_, δ): 3.87 (s, 3H, OCH_3_), 3.97 (s, 3H, OCH_3_), 7.13 (d, 1H, *J* = 2.0 Hz, H-3_furan_), 7.52–7.56 (m, 3H, H-3_quinoline_, H-5_quinoline_ and H-6_quinoline_), 7.86 (d, 1H, *J* = 2.0 Hz, H-2_furan_), 8.04 (s, 1H, H-8_quinoline_), 8.18 (d, 1H, *J* = 7.6 Hz, H-2_quinoline_), 8.53 (s, 1H, H-4_pyridine_), 8.69 (s, 1H, H-2_pyridine_), 9.42 (s, 2H, NH_2_ exchangeable with D_2_O), 12.52 (s, 1H, OH exchangeable with D_2_O). ^13^C NMR (75 MHz, DMSO-*d*_6_, δ): 58.5 (OMe), 59.3 (OMe), 102.5 (C3a′), 106.4 (C3′), 109.3 (C3a), 112.5 (C5′), 122.0 (Ar-C), 122.6 (Ar-C), 123.3 (Ar-C), 123.7 (C7′), 124.6 (Ar-C), 125.1 (Ar-C), 126.4 (Ar-C), 127.8 (Ar-C), 128.6 (C4′), 134.5 (C8a_quinoline_), 139.5 (C-7a), 140.5 (C4_quinoline_), 143.4 (C2 _quinoline_), 144.5 (C-4), 145.2 (C-3), 147.4 (C-2′), 148.2 (C-6), 150.5 (C6′), 152.6 (C7a′), 192.2 (C=O_ketone_). Mass spectrum, *m/z* (*I*_r_ %): 515/517 (M^+^/M+2, 100/33), 354 (68), 312 (32), 221 (54), 162/164 (69/23), 134 (11), 117 (19), 94 (42), 77 (26), 64 (15). Anal. Calcd for C_26_H_18_ClN_5_O_5_ (515.90): C, 60.53; H, 3.52; N, 13.57%. Found: C, 60.41; H, 3.39; N, 13.52%.

5-Amino-3-[(6-hydroxy-4,7-dimethoxy-1-benzofuran-5-yl)carbonyl]pyrido[3,2-*e*] [1,2,4]triazolo[4,3-*a*]pyrimidine (**6**)

A mixture of compound **1** (0.72 g, 2 mmol) and 3-amino-1,2,4-triazole (0.16 g, 2 mmol), in DMF (10 mL) containing TEA (0.1 mL), was heated under reflux for 4 h. After cooling, the yellow crystals deposited were filtered and recrystallized from *iso*-butanol, mp > 300 °C, yield (0.59 g, 72%). IR (KBr, cm^−1^): 3406 (OH), 3348, 3265 (NH_2_), 1649 (C=O), 1615 (C=N), 1594 (C=C). ^1^H NMR (400 MHz, DMSO-*d*_6_, δ): 3.84 (s, 3H, OCH_3_), 3.92 (s, 3H, OCH_3_), 7.11 (d, 1H, *J* = 2.0 Hz, H-3_furan_), 7.92 (d, 1H, *J* = 2.0 Hz, H-2_furan_), 8.43 (s, 1H, H-4_pyridine_), 8.55 (s, 1H, H-2_pyridine_), 8.97 (s, 1H, H-3_triazole_), 9.50 (s, 2H, NH_2_ exchangeable with D_2_O), 12.33 (s, 1H, OH exchangeable with D_2_O). ^13^C NMR (100 MHz, DMSO-*d*_6_, δ): 58.0 (OCH_3_), 59.2 (OCH_3_), 102.3 (C5′), 104.3 (C4a), 106.7 (C3′), 109.3 (C3a′), 122.4 (C7′), 128.2 (C3), 129.6 (C4′), 137.3 (C9), 138.4 (C4), 139.3 (C2), 144.9 (C5), 145.2 (C2′), 148.5 (C10a), 149.7 (C6a), 151.6 (C6′), 152.8 (C7a′), 192.3 (C=O_ketone_). Mass spectrum, *m/z* (*I*_r_ %): 406 (M^+^, 100), 350 (67), 320 (39), 288 (32), 221 (51), 185 (24), 148 (28), 134 (15), 118 (11), 94 (47), 77 (33), 64 (10). Anal. Calcd for C_19_H_14_N_6_O_5_ (406.35): C, 56.16; H, 3.47; N, 20.68%. Found: C, 55.96; H, 3.44; N, 20.39%.

5-Amino-7-[(6-hydroxy-4,7-dimethoxy-1-benzofuran-5-yl)carbonyl]pyrido[3,2-*e*] [1,2,4]tetrazolo[1,5-*a*]pyrimidine (**7**)

A mixture of compound **1** (0.72 g, 2 mmol) and 5-amino-1*H*-tetrazole (0.16 g, 2 mmol), in DMF (10 mL) containing TEA (0.1 mL), was heated under reflux for 4 h. After cooling, the yellow crystals so formed were filtered and recrystallized from *iso*-butanol, mp > 300 °C, yield (0.57 g, 70%). IR (KBr, cm^−1^): 3403 (OH), 3369, 3287 (NH_2_), 1644 (C=O), 1611 (C=N), 1590 (C=C). ^1^H NMR (400 MHz, DMSO-*d*_6_, δ): 3.86 (s, 3H, OCH_3_), 4.03 (s, 3H, OCH_3_), 7.19 (d, 1H, *J* = 2.0 Hz, H-3_furan_), 7.95 (d, 1H, *J* = 2.0 Hz, H-2_furan_), 8.52 (s, 1H, H-4_pyridine_), 8.61 (s, 1H, H-2_pyridine_), 9.26 (s, 2H, NH_2_ exchangeable with D_2_O), 12.40 (s, 1H, OH exchangeable with D_2_O). ^13^C NMR (100 MHz, DMSO-*d*_6_, δ): 58.5 (OCH_3_), 59.1 (OCH_3_), 101.8 (C5′), 105.1 (C4a), 106.2 (C3′), 110.3 (C3a′), 123.1 (C7′), 128.5 (C3), 129.8 (C4′), 138.5 (C4), 138.8 (C2), 144.2 (C5), 145.1 (C2′), 148.1 (C10a), 149.2 (C6a), 151.1 (C6′), 152.7 (C7a′), 192.4 (C=O_ketone_). Mass spectrum, *m/z* (*I*_r_ %): 407 (M^+^, 48), 349 (30), 304 (25), 274 (19), 221 (36), 192 (16), 171 (14), 159 (22), 133 (18), 117 (14), 93 (100), 77 (64), 64 (24). Anal. Calcd for C_18_H_13_N_7_O_5_ (407.34): C, 53.07; H, 3.22; N, 24.07%. Found: C, 52.83; H, 3.14; N, 23.95%.

5-Amino-3-[(6-hydroxy-4,7-dimethoxy-1-benzofuran-5-yl)carbonyl]-9-methyl-10*H*-pyrido[3′,2′:5,6]pyrimido[2,1-*c*][1,2,4]triazin-10-one (**9**)

A mixture of compound **1** (0.72 g, 2 mmol) and 3-amino-6-methyl-1,2,4-triazin-5(4*H*)-one (**8**) (0.25 g, 2 mmol), in DMF (10 mL) containing TEA (0.1 mL), was heated under reflux for 4 h. After cooling, the pale-brown crystals so formed were filtered and recrystallized from AcOH/H_2_O, mp > 300 °C, yield (0.62 g, 69%). IR (KBr, cm^−1^): 3405 (OH), 3372, 3296 (NH_2_), 1692 (C=O_triazine_), 1654 (C=O), 1604 (C=N), 1587 (C=C). ^1^H NMR (400 MHz, DMSO-*d*_6_, δ): 2.18 (s, 3H, CH_3 triazine_), 3.82 (s, 3H, OCH_3_), 3.91 (s, 3H, OCH_3_), 7.25 (d, 1H, *J* = 2.4 Hz, H-3_furan_), 7.85 (d, 1H, *J* = 2.4 Hz, H-2_furan_), 8.49 (s, 1H, H-4_pyridine_), 8.58 (s, 1H, H-2_pyridine_), 9.32 (s, 2H, NH_2_ exchangeable with D_2_O), 12.40 (s, 1H, OH exchangeable with D_2_O). ^13^C NMR (100 MHz, DMSO-*d*_6_, δ): 17.3 (CH_3_), 58.8 (OCH_3_), 59.3 (OCH_3_), 103.4 (C5′), 104.9 (C4a), 106.6 (C3′), 110.2 (C3a′), 123.0 (C7′), 129.3 (C4′), 130.0 (C3), 135.4 (C9), 138.5 (C4), 139.1 (C2), 143.6 (C5), 145.0 (C11a), 146.2 (C2′), 148.5 (C6a), 150.5 (C6′), 152.7 (C7a′), 166.2 (C=O_triazine_), 193.6 (C=O_ketone_). Mass spectrum, *m/z* (*I*_r_ %): 448 (M^+^, 68), 418 (100), 388 (32), 346 (29), 227 (17), 194 (15), 159 (17), 133 (22), 118 (13), 92 (36), 77 (32), 64 (14). Anal. Calcd for C_21_H_16_N_6_O_6_ (448.39): C, 56.25; H, 3.60; N, 18.74%. Found: C, 56.03; H, 3.47; N, 18.65%.

5-Amino-3-[(6-hydroxy-4,7-dimethoxy-1-benzofuran-5-yl)carbonyl]benzo[4,5] imidazo[1,2-*a*]pyrido[3,2-*e*]pyrimidine (**10**)

A mixture of compound **1** (0.72 g, 2 mmol) and 2-aminobenzimidazole (0.27 g, 2 mmol), in DMF (10 mL) containing TEA (0.1 mL), was heated under reflux for 4 h. After cooling, the yellow crystals deposited were filtered and recrystallized from AcOH, mp > 300 °C, yield (0.65 g, 71%). IR (KBr, cm^−1^): 3417 (OH), 3383, 3268 (NH_2_), 1648 (C=O), 1607 (C=N), 1582 (C=C). ^1^H NMR (400 MHz, DMSO-*d*_6_, δ): 3.84 (s, 3H, OCH_3_), 3.98 (s, 3H, OCH_3_), 7.15 (d, 1H, *J* = 2.4 Hz, H-3_furan_), 7.37–7.43 (m, 2H, Ar-H), 7.48–7.53 (m, 2H, Ar-H), 7.86 (d, 1H, *J* = 2.4 Hz, H-2_furan_), 8.42 (s, 1H, H-4_pyridine_), 8.53 (s, 1H, H-2_pyridine_), 9.52 (s, 2H, NH_2_ exchangeable with D_2_O), 12.33 (s, 1H, OH exchangeable with D_2_O). ^13^C NMR (100 MHz, DMSO-*d*_6_, δ): 58.5 (OCH_3_), 59.7 (OCH_3_), 102.1 (C5′), 104.8 (C4a), 106.2 (C3′), 110.5 (C3a′), 120.3 (Ar-C), 121.1 (Ar-C), 122.8 (C7′), 124.1 (Ar-C), 125.2 (Ar-C), 126.1 (Ar-C), 129.6 (C4′), 130.3 (C3), 134.3 (Ar-C), 138.3 (C4), 139.7 (C2), 142.9 (C-13a), 144.7 (C5), 146.3 (C2′), 148.1 (C6a), 150.3 (C6′), 151.9 (C7a′), 192.3 (C=O_ketone_). Mass spectrum, *m/z* (*I*_r_ %): 455 (M^+^, 100), 395 (59), 340 (46), 234 (21), 220 (35), 190 (24), 161 (32), 134 (26), 117 (10), 94 (56), 77 (42), 65 (17). Anal. Calcd for C_24_H_17_N_5_O_5_ (455.42): C, 63.29; H, 3.76; N, 15.38%. Found: C, 63.14; H, 3.52; N, 15.31%.

4-Amino-6-[(6-hydroxy-4,7-dimethoxy-1-benzofuran-5-yl)carbonyl]-2-oxo-1,2-dihydro-1,8-naphthyridine-3-carbonitrile (**11**)

A mixture of compound **1** (0.72 g, 2 mmol) and cyanoacetamide (0.16 g, 2 mmol) in DMF (10 mL) containing TEA (0.1 mL), was heated under reflux for 4 h. After cooling, the yellow crystals so formed were filtered and recrystallized from DMF/H_2_O, mp > 300 °C, yield (0.59 g, 73%). IR (KBr, cm^−1^): 3411 (OH), 3385, 3316, 3274 (NH_2_, NH), 2224 (C≡N), 1681 (C=O_pyridine_), 1650 (C=O), 1608 (C=N), 1584 (C=C). ^1^H NMR (400 MHz, DMSO-*d*_6_, δ): 3.89 (s, 3H, OCH_3_), 3.97 (s, 3H, OCH_3_), 7.23 (d, 1H, *J* = 2.4 Hz, H-3_furan_), 7.91 (d, 1H, *J* = 2.4 Hz, H-2_furan_), 8.46 (s, 1H, H-4_pyridine_), 8.52 (s, 1H, H-2_pyridine_), 9.32 (s, 2H, NH_2_ exchangeable with D_2_O), 11.04 (s, 1H, NH exchangeable with D_2_O), 12.64 (s, 1H, OH exchangeable with D_2_O). ^13^C NMR (100 MHz, DMSO-*d*_6_, δ): 59.0 (OCH_3_), 60.2 (OCH_3_), 87.1 (C3), 102.7 (C5′), 105.8 (C3′), 111.2 (C3a′), 116.3 (C≡N), 122.8 (C7′), 123.2 (C4a), 128.4 (C6), 129.8 (C4′), 138.2 (C5), 140.0 (C7), 144.7 (C4), 146.1 (C2′), 148.8 (C8a), 150.8 (C6′), 152.3 (C7a′), 169.5 (C2 as C=O_naphthyridine_), 193.2 (C=O_ketone_). Mass spectrum, *m/z* (*I*_r_ %): 406 (M^+^, 100), 340 (25), 256 (20), 221 (49), 172 (38), 133 (14), 117 (23), 93 (46), 77 (28), 64 (13). Anal. Calcd for C_20_H_14_N_4_O_6_ (406.35): C, 59.12; H, 3.47; N, 13.79%. Found: C, 59.06; H, 3.30; N, 13.58%.

4-Amino-1-benzyl-6-[(6-hydroxy-4,7-dimethoxy-1-benzofuran-5-yl)carbonyl]-2-oxo-1,2-dihydro-1,8-naphthyridine-3-carbonitrile (**12**)

A mixture of compound **1** (0.72 g, 2 mmol) and *N*-benzylcyanoacetamide (0.32 g, 2 mmol), in DMF (10 mL) containing TEA (0.1 mL), was heated under reflux for 4 h. After cooling, the yellow crystals deposited were filtered and recrystallized from AcOH, mp > 300 °C, yield (0.68 g, 68%). IR (KBr, cm^−1^): 3415 (OH), 3371, 3288 (NH_2_), 2221 (C≡N), 1686 (C=O_pyridine_), 1657 (C=O), 1613 (C=N), 1579 (C=C). ^1^H NMR (400 MHz, DMSO-*d*_6_, δ): 3.08 (s, 2H, CH_2_), 3.83 (s, 3H, OCH_3_), 3.97 (s, 3H, OCH_3_), 7.18 (d, 1H, *J* = 2.0 Hz, H-3_furan_), 7.54–7.66 (m, 5H, Ar-H), 7.93 (d, 1H, *J* = 2.4 Hz, H-2_furan_), 8.42 (s, 1H, H-4_pyridine_), 8.50 (s, 1H, H-2_pyridine_), 9.28 (s, 2H, NH_2_ exchangeable with D_2_O), 12.32 (s, 1H, OH exchangeable with D_2_O). ^13^C NMR (100 MHz, DMSO-*d*_6_, δ): 29.4 (NCH_2_), 58.3 (OCH_3_), 59.5 (OCH_3_), 88.4 (C3), 103.5 (C5′), 106.2 (C3′), 111.4 (C3a′), 116.6 (C≡N), 123.1 (C7′), 125.3 (C4a), 125.6 (Ar-C), 127.0 (Ar-C), 128.6 (C6), 129.2 (C4′), 130.6 (Ar-C), 134.1 (Ar-C), 138.6 (C5), 139.2 (C7), 144.5 (C4), 146.6 (C2′), 149.2 (C8a), 150.8 (C6′), 152.5 (C7a′), 169.1 (C2 as C=O_naphthyridine_), 194.1 (C=O_ketone_). Mass spectrum, *m/z* (*I*_r_ %): 496 (M^+^, 59), 466 (52), 375 (46), 309 (47), 242 (32), 220 (64), 194 (26), 159 (21), 134 (15), 118 (16), 91 (100), 77 (57), 64 (23). Anal. Calcd for C_27_H_20_N_4_O_6_ (496.47): C, 65.32; H, 4.06; N, 11.29%. Found: C, 65.14; H, 4.01; N, 11.15%.

5-Amino-3-[(6-hydroxy-4,7-dimethoxy-1-benzofuran-5-yl)carbonyl]-benzo[4,5]imidazo[1,2-*a*][1,8]naphthyridine-6-carbonitrile (**13**)

A mixture of compound **1** (0.72 g, 2 mmol) and 1*H*-benzimidazol-2-ylacetonitrile (0.31 g, 2 mmol), in DMF (10 mL) containing TEA (0.1 mL), was heated under reflux for 4 h. After cooling, the pale-brown crystals deposited were filtered and recrystallized from DMF, mp > 300 °C, yield (0.71 g, 74%). IR (KBr, cm^−1^): 3404 (OH), 3361, 3279 (NH_2_), 2226 (C≡N), 1651 (C=O), 1612 (C=N), 1576 (C=C). ^1^H NMR (400 MHz, DMSO-*d*_6_, δ): 3.86 (s, 3H, OCH_3_), 3.93 (s, 3H, OCH_3_), 7.13 (d, 1H, *J* = 2.0 Hz, H-3_furan_), 7.37–7.42 (m, 4H, Ar-H), 7.88 (d, 1H, *J* = 2.0 Hz, H-2_furan_), 8.39 (s, 1H, H-4_pyridine_), 8.57 (s, 1H, H-2_pyridine_), 9.41 (s, 2H, NH_2_ exchangeable with D_2_O), 12.33 (s, 1H, OH exchangeable with D_2_O). ^13^C NMR (75 MHz, DMSO-*d*_6_, δ): 59.1 (OMe), 60.2 (OMe), 87.3 (C-6), 102.9 (C3a′), 105.8 (C3′), 108.6 (C-9), 112.5 (C5′), 113.1 (C-7a), 117.2 (C≡N), 120.7 (Ar-C), 122.8 (C7′), 124.1 (Ar-C), 124.7 (Ar-C), 128.9 (Ar-C), 129.8 (C4′), 130.7 (Ar-C), 132.2 (Ar-C), 138.7 (C-11a), 142.3 (C-8), 143.2 (C-10), 145.1 (C-7), 147.2 (C2′), 148.0 (C-5a), 151.9 (C6′), 152.4 (C7a′), 191.3 (C=O_ketone_). Mass spectrum, *m/z* (*I*_r_ %): 479 (M^+^, 100), 419 (64), 353 (47), 313 (43), 258 (24), 221 (36), 192 (18), 161 (15), 133 (12), 117 (19), 94 (68), 77 (44), 64 (19). Anal. Calcd for C_26_H_17_N_5_O_5_ (479.44): C, 65.13; H, 3.57; N, 14.61%. Found: C, 64.96; H, 3.40; N, 14.39%.

4-Amino-6-[(6-hydroxy-4,7-dimethoxy-1-benzofuran-5-yl)carbonyl]-1,2-dihydro-3*H*-pyrazolo[3,4-*b*][1,8]naphthyridin-3-one (**14**)

A mixture of compound **1** (0.72 g, 2 mmol) and 5-amino-2,4-dihydro-3*H*-pyrazol-3-one (0.20 g, 2 mmol), in DMF (10 mL) containing TEA (0.1 mL), was heated under reflux for 4 h. After cooling, the pale-brown crystals deposited were filtered and recrystallized from DMF, mp > 300 °C, yield (0.64 g, 76%). IR (KBr, cm^−1^): 3407 (OH), 3376, 3338, 3296 (NH_2_, 2NH), 1667 (C=O_pyrazole_), 1646 (C=O), 1605 (C=N), 1582 (C=C). ^1^H NMR (400 MHz, DMSO-*d*_6_, δ): 3.87 (s, 3H, OCH_3_), 4.03 (s, 3H, OCH_3_), 7.16 (d, 1H, *J* = 2.0 Hz, H-3_furan_), 7.93 (d, 1H, *J* = 2.0 Hz, H-2_furan_), 8.50 (s, 1H, H-4_pyridine_), 8.69 (s, 1H, H-2_pyridine_), 9.39 (s, 2H, NH_2_ exchangeable with D_2_O), 10.44 (s, 1H, NH exchangeable with D_2_O), 11.28 (s, 1H, NH exchangeable with D_2_O), 12.48 (s, 1H, OH exchangeable with D_2_O). ^13^C NMR (100 MHz, DMSO-*d*_6_, δ): 59.4 (OCH_3_), 59.9 (OCH_3_), 103.4 (C5′), 105.5 (C3a), 106.7 (C3′), 111.6 (C3a′), 112.4 (C5a), 122.6 (C7′), 128.3 (C6), 129.2 (C4′), 137.4 (C5), 138.1 (C7), 143.0 (C9a), 145.2 (C4), 146.2 (C2′), 148.4 (C8a), 150.7 (C6′), 152.5 (C7a′), 165.5 (C=O_pyrazolone_), 192.0 (C=O_ketone_). Mass spectrum, *m/z* (*I*_r_ %): 421 (M^+^, 100), 378 (47), 318 (56), 278 (27), 221 (38), 201 (41), 172 (30), 157 (14), 133 (17), 118 (21), 93 (48), 77 (36), 65 (12). Anal. Calcd for C_20_H_15_N_5_O_6_ (421.36): C, 57.01; H, 3.59; N, 16.62%. Found: C, 56.86; H, 3.47; N, 16.50%.

4-Amino-6-(6-hydroxy-4,7-dimethoxy-1-benzofuran-5-yl)carbonyl]-3-methyl-1*H*-pyrazolo[3,4-*b*][1,8]naphthyridine (**15**)

A mixture of compound **1** (0.72 g, 2 mmol) and 5-amino-3-methyl-1*H*-pyrazole (0.20 g, 2 mmol), in DMF (10 mL) containing TEA (0.1 mL), was heated under reflux for 4 h. After cooling, the yellow crystals so formed were filtered and recrystallized from DMF/H_2_O, mp > 300 °C, yield (0.66 g, 79%). IR (KBr, cm^−1^): 3412 (OH), 3359, 3281 (NH_2_), 1648 (C=O), 1617 (C=N), 1589 (C=C). ^1^H NMR (400 MHz, DMSO-*d*_6_, δ): 2.42 (s, 3H, CH_3 pyrazole_), 3.92 (s, 3H, OCH_3_), 4.00 (s, 3H, OCH_3_), 7.22 (d, 1H, *J* = 2.4 Hz, H-3_furan_), 7.96 (d, 1H, *J* = 2.4 Hz, H-2_furan_), 8.52 (s, 1H, H-4_pyridine_), 8.64 (s, 1H, H-2_pyridine_), 9.32 (s, 2H, NH_2_ exchangeable with D_2_O), 10.36 (s, 1H, NH exchangeable with D_2_O), 12.41 (s, 1H, OH exchangeable with D_2_O). ^13^C NMR (100 MHz, DMSO-*d*_6_, δ): 18.6 (CH_3_), 59.2 (OCH_3_), 60.0 (OCH_3_), 102.8 (C5′), 105.7 (C3a), 106.1 (C3′), 111.5 (C3a′), 113.4 (C5a), 122.4 (C7′), 125.4 (C6), 129.6 (C4′), 135.3 (C3), 137.2 (C6), 138.1 (C8), 142.6 (C9a), 143.0 (C4), 145.9 (C2′), 148.5 (C8a), 150.8 (C6′), 153.1 (C7a′), 191.6 (C=O_ketone_). Mass spectrum, *m/z* (*I*_r_ %): 419 (M^+^, 68), 389 (100), 319 (42), 278 (37), 220 (51), 198 (24), 157 (20), 133 (26), 118 (27), 92 (39), 77 (28), 64 (17). Anal. Calcd for C_21_H_17_N_5_O_5_ (419.39): C, 60.14; H, 4.09; N, 16.70%. Found: C, 59.93; H, 3.84; N, 16.59%.

5-Amino-7-[(6-hydroxy-4,7-dimethoxy-1-benzofuran-5-yl)carbonyl] pyrimido[4,5-*b*][1,8]naphthyridine-2,4(1*H*,3*H*)-dione (**16**)

A mixture of compound **1** (0.72 g, 2 mmol) and 6-amino-2,3-dihydro pyrimidin-2,4(1*H*,3*H*)-dione (0.27 g, 2 mmol), in DMF (10 mL) containing TEA (0.1 mL), was heated under reflux for 4 h. After cooling, the yellow crystals deposited were filtered and recrystallized from AcOH/H_2_O, mp > 300 °C, yield (0.67 g, 75%). IR (KBr, cm^−1^): 3407 (OH), 3384, 3297, 3227 (NH_2_, 2NH), 1679 (2C=O_pyrimidine_), 1656 (C=O), 1614 (C=N), 1583 (C=C). ^1^H NMR (400 MHz, DMSO-*d*_6_, δ): 3.92 (s, 3H, OCH_3_), 3.98 (s, 3H, OCH_3_), 7.13 (d, 1H, *J* = 2.4 Hz, H-3_furan_), 7.88 (d, 1H, *J* = 2.4 Hz, H-2_furan_), 8.34 (s, 1H, H-4_pyridine_), 8.72 (s, 1H, H-2_pyridine_), 9.58 (s, 2H, NH_2_ exchangeable with D_2_O), 10.33 (s, 1H, NH exchangeable with D_2_O), 10.70 (s, 1H, NH exchangeable with D_2_O), 12.45 (s, 1H, OH exchangeable with D_2_O). ^13^C NMR (100 MHz, DMSO-*d*_6_, δ): 58.7 (OCH_3_), 59.6 (OCH_3_), 102.5 (C5′), 104.0 (C4a), 107.4 (C3′), 109.2 (C3a′), 111.2 (C5a), 122.9 (C7′), 127.6 (C3), 129.3 (C4′), 137.8 (C6), 139.1 (C8), 144.3 (C10a), 144.9 (C5), 147.4 (C2′), 148.1 (C9a), 150.5 (C6′), 152.7 (C7a′), 165.1 (C2 as C=O_pyrimidine_), 167.4 (C4 as C=O_pyrimidine_), 192.8 (C=O_ketone_). Mass spectrum, *m/z* (*I*_r_ %): 449 (M^+^, 100), 391 (68), 346 (39), 305 (43), 262 (52), 229 (41), 185 (38), 157 (19), 134 (25), 117 (16), 94 (31), 77 (28), 64 (10). Anal. Calcd for C_21_H_15_N_5_O_7_ (449.37): C, 56.13; H, 3.36; N, 15.58%. Found: C, 56.02; H, 3.21; N, 15.37%.

5-Amino-7-[(6-hydroxy-4,7-dimethoxy-1-benzofuran-5-yl)carbonyl]-2-thioxo-2,3-dihydropyrimido[4,5-*b*][1,8]naphthyridin-4(1*H*)-one (**17**)

A mixture of compound **1** (0.72 g, 2 mmol) and 6-amino-2-thioxo-2,3-dihydropyrimidin-4(1*H*)-one (0.29 g, 2 mmol), in DMF (10 mL) containing TEA (0.1 mL), was heated under reflux for 4 h. After cooling, the canary yellow crystals so formed were filtered and recrystallized from AcOH, mp > 300 °C, yield (0.73 g, 78%). IR (KBr, cm^−1^): 3401 (OH), 3370, 3284, 3216 (NH_2_, 2NH), 1673 (C=O_pyrimidine_), 1652 (C=O), 1619 (C=N), 1587 (C=C), 1226 (C=S). ^1^H NMR (400 MHz, DMSO-*d*_6_, δ): 3.90 (s, 3H, OCH_3_), 3.98 (s, 3H, OCH_3_), 7.08 (d, 1H, *J* = 2.8 Hz, H-3_furan_), 7.87 (d, 1H, *J* = 2.8 Hz, H-2_furan_), 8.53 (s, 1H, H-4_pyridine_), 8.67 (s, 1H, H-2_pyridine_), 9.34 (s, 2H, NH_2_ exchangeable with D_2_O), 11.42 (s, 1H, NH exchangeable with D_2_O), 11.78 (s, 1H, NH exchangeable with D_2_O), 12.64 (s, 1H, OH exchangeable with D_2_O). ^13^C NMR (100 MHz, DMSO-*d*_6_, δ): 58.5 (OCH_3_), 59.7 (OCH_3_), 103.4 (C5′), 105.3 (C4a), 107.2 (C3′), 109.5 (C3a′), 112.4 (C5a), 123.1 (C7′), 126.9 (C3), 128.7 (C4′), 136.4 (C6), 137.6 (C8), 142.1 (C10a), 143.2 (C5), 146.3 (C2′), 147.8 (C9a), 151.2 (C6′), 152.5 (C7a′), 168.9 (C4 as C=O_pyrimidine_), 186.3 (C2 as C=S_pyrimidine_), 194.8 (C=O_ketone_). Mass spectrum, *m/z* (*I*_r_ %): 465 (M^+^, 78), 407 (58), 348 (61), 318 (55), 263 (47), 221 (56), 173 (16), 159 (26), 133 (29), 118 (32), 92 (100), 77 (46), 64 (13). Anal. Calcd for C_21_H_15_N_5_O_6_S (465.44): C, 54.19; H, 3.25; N, 15.05; S, 6.89%. Found: C, 53.85; H, 3.17; N, 14.93; S, 6.81%.

5-Amino-7-[(6-hydroxy-4,7-dimethoxy-1-benzofuran-5-yl)carbonyl]1,3-dimethyl-pyrimido[4,5-*b*][1,8]naphthyridine-2,4(1*H*,3*H*)-dione (**18**)

A mixture of compound **1** (0.72 g, 2 mmol) and 6-amino-1,3-dimethylpyrimidine- 2,4(1*H*,3*H*)-dione (0.31 g, 2 mmol), in DMF (10 mL) containing TEA (0.1 mL) was heated under reflux for 4 h. After cooling, the pale-yellow crystals so formed were filtered and recrystallized from AcOH/H_2_O, mp > 300 °C, yield (0.74 g, 77%). IR (KBr, cm^−1^): 3402 (OH), 3376, 3283 (NH_2_), 1676 (2C=O_pyrimidine_), 1658 (C=O), 1610 (C=N), 1588 (C=C). ^1^H NMR (400 MHz, DMSO-*d*_6_, δ): 3.06 (s, 3H, NCH_3_), 3.17 (s, 3H, NCH_3_), 3.92 (s, 3H, OCH_3_), 4.04 (s, 3H, OCH_3_), 7.25 (d, 1H, *J* = 2.0 Hz, H-3_furan_), 7.97 (d, 1H, *J* = 2.0 Hz, H-2_furan_), 8.52 (s, 1H, H-4_pyridine_), 8.68 (s, 1H, H-2_pyridine_), 9.37 (s, 2H, NH_2_ exchangeable with D_2_O), 12.52 (s, 1H, OH exchangeable with D_2_O). ^13^C NMR (100 MHz, DMSO-*d*_6_, δ): 28.9 (NCH_3_), 30.0 (NCH_3_), 58.9 (OCH_3_), 59.7 (OCH_3_), 102.6 (C5′), 103.9 (C4a), 106.7 (C3′), 109.8 (C3a′), 111.1 (C5a), 123.2 (C7′), 126.8 (C3), 129.0 (C4′), 137.1 (C6), 139.3 (C8), 143.6 (C10a), 144.7 (C5), 146.2 (C2′), 148.3 (C9a), 151.2 (C6′), 151.8 (C7a′), 165.5 (C2 as C=O_pyrimidine_), 168.1 (C4 as C=O_pyrimidine_), 192.7 (C=O_ketone_). Mass spectrum, *m/z* (*I*_r_ %): 477 (M^+^, 100), 416 (72), 375 (64), 333 (47), 256 (58), 221 (65), 194 (37), 173 (46), 158 (25), 133 (29), 118 (21), 94 (22), 77 (18), 64 (9). Anal. Calcd for C_23_H_19_N_5_O_7_ (477.43): C, 57.86; H, 4.01; N, 14.67%. Found: C, 57.64; H, 3.95; N, 14.48%.

## 4. Conclusions

In the current study, the recently synthesized 2-chloro-5-[(6-hydroxy-4,7- dimethoxy-1-benzofuran-5-yl)carbonyl]pyridine-3-carbonitrile (**1**) was efficiently utilized as a building block for the construction of various heterocyclic systems. Linear and angular annulated pyridines linked to the (6-hydroxy-4,7-dimethoxy-1-benzofuran-5-yl)carbonyl were efficiently synthesized through the reaction of starting precursor **1** with binucleophilic reagents. All the synthesized compounds showed a remarkable effect against yeast and fungus strains, while compounds **4**, **5**, **7**, **9**, **11**, **12** and **16**–**18** exhibited significant inhibitory effects against all tested microorganisms.

## Figures and Tables

**Table 1:** Antimicrobial estimation, in vitro, for the prepared compounds **1**–**18** by disc diffusion measurement.

No.	Zone Diameter (mm) * (% with Respect to Reference Drug)											
	Gram-Positive Bacteria				Gram-Negative Bacteria				Yeasts and Fungi			
Compd. No.	*S. aureus*		*B. subtilis*		*S. typhimurium*		*E. coli*		*C. albicans*		*A. fumigatus*	
	1000 μg/mL	500 μg/mL	1000 μg/mL	500 μg/mL	1000 μg/mL	500 μg/mL	1000 μg/mL	500 μg/mL	1000 μg/mL	500 μg/mL	1000 μg/mL	500 μg/mL
**1**	40%	38%	49%	48%	53%	50%	45%	44%	69%	71%	70%	73%
**4**	77%	77%	69%	72%	69%	68%	71%	74%	86%	75%	73%	77%
**5**	83%	81%	77%	76%	75%	68%	74%	70%	80%	75%	70%	69%
**6**	46%	50%	51%	52%	47%	46%	39%	41%	71%	64%	77%	73%
**7**	80%	77%	74%	76%	75%	71%	74%	70%	86%	79%	77%	81%
**9**	86%	88%	83%	84%	75%	71%	68%	74%	83%	75%	73%	73%
**10**	57%	58%	49%	48%	42%	39%	45%	52%	71%	68%	73%	77%
**11**	80%	81%	71%	72%	78%	75%	68%	70%	77%	71%	68%	69%
**12**	71%	69%	69%	72%	72%	71%	74%	78%	74%	68%	78%	77%
**13**	49%	42%	46%	40%	53%	43%	42%	41%	69%	71%	70%	69%
**14**	54%	54%	43%	44%	58%	50%	47%	44%	74%	71%	77%	81%
**15**	51%	50%	49%	48%	50%	46%	39%	41%	77%	75%	68%	73%
**16**	77%	77%	74%	72%	78%	68%	66%	67%	74%	71%	77%	81%
**17**	89%	85%	71%	72%	72%	64%	71%	70%	77%	75%	68%	73%
**18**	83%	83%	80%	76%	75%	71%	68%	71%	71%	68%	77%	77%
**S**	100%	100%	100%	100%	100%	100%	100%	100%	100%	100%	100%	100%

* Calculated from 3 times. **S**: Standard antibiotics, which are Cycloheximide for fungus and yeast, Chloramphencol for Gram-positive bacteria, and Cephalothin for Gram-negative bacteria.

## Data Availability

Data are contained within the article and Supplementary Materials.

## Supplementary Materials

The following supporting information can be downloaded at: https://www.mdpi.com/article/10.3390/molecules29184496/s1. Supporting Materials: (A) Copies of 1H-NMR, 13C-NMR and mass spectral data for the synthesized compounds and (B) Antimicrobial efficiency of the synthesized compounds with respected to references drugs.
