# Supplementary material for: Linear and Angular Heteroannulated Pyridines Tethered 6-Hydroxy-4,7-Dimethoxybenzofuran: Synthesis and Antimicrobial Activity
Source: Molecules. 2024 Sep 22;29(18):4496. doi: 10.3390/molecules29184496 (PMC11434407; doi:10.3390/molecules29184496)
Supplement: Supplementary file 1 [file molecules-29-04496-s001.zip › molecules-3223079-supplementary.pdf]

# Linear and Angular Heteroannulated Pyridines Tethered 6-Hydroxy-4,7-Dimethoxybenzofuran: Synthesis and Antimicrobial Activity

**Najla A. Alshaye [1], Al-Shimaa Badran [2] and Magdy A. Ibrahim [2,*]**

*1) Department of Chemistry, College of Science, Princess Nourah bint Abdulrahman University, P.O. Box 84428, Riyadh 11671, Saudi Arabia; naalshaye@pnu.edu.sa*

*2) Department of Chemistry, Faculty of Education, Ain Shams University, Roxy, Cairo 11711, Egypt; elshimaabadran@edu.asu.edu.eg*

**E-mail: magdy_ahmed1977@yahoo.com*, Tel.: +2-01007887204

## (A) Copies of $^1$H-NMR, $^{13}$C-NMR and mass spectral data for the synthesized compounds:

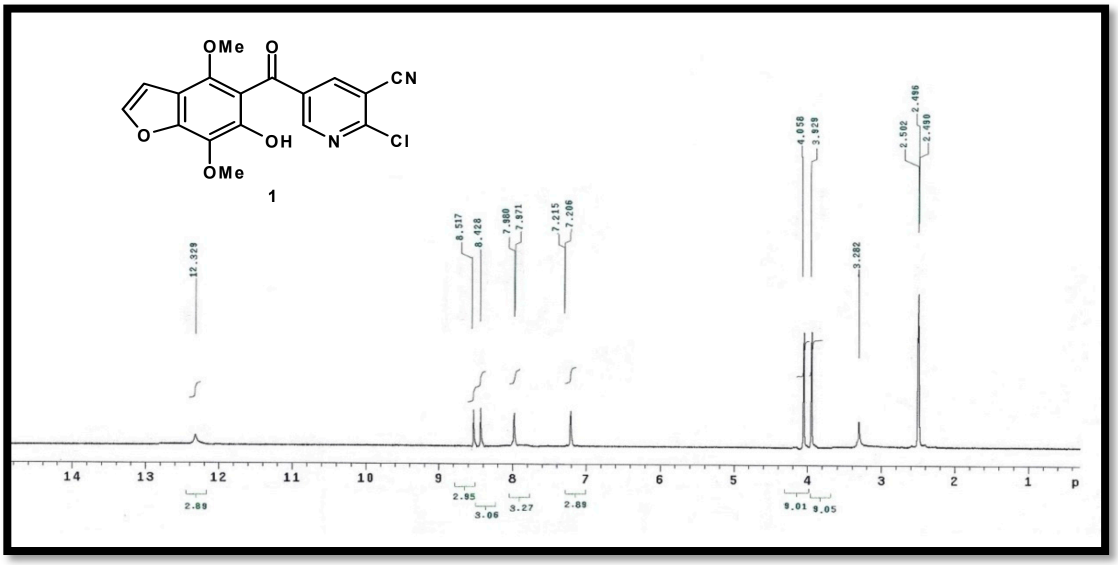


Figure S1 (DMSO-$d_6$, 400 MHz): $^1$H NMR spectrum of compound 1

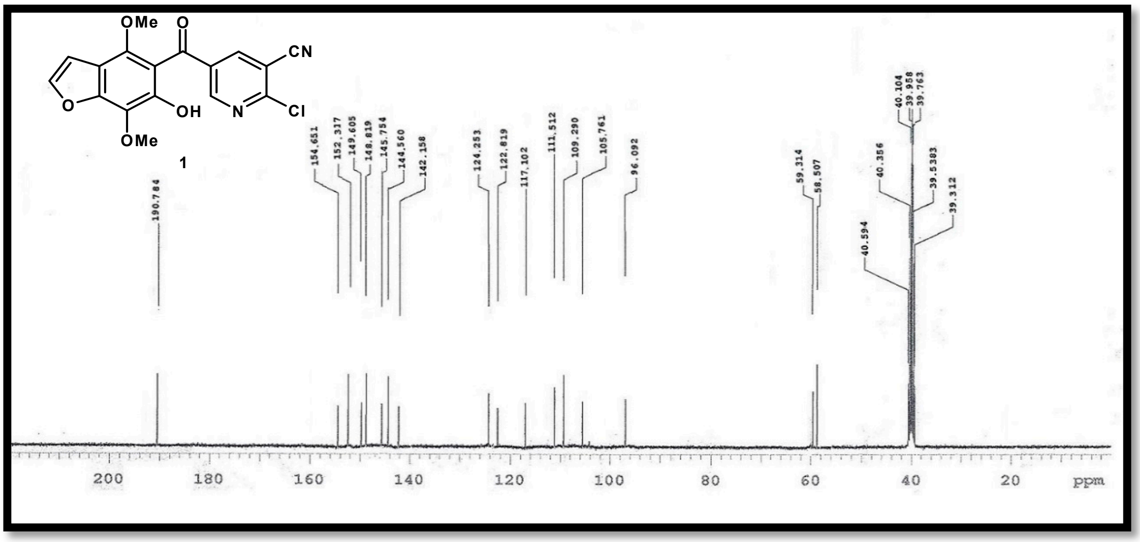


Figure S2 (DMSO-$d_6$, 100 MHz): $^{13}$C NMR spectrum of compound 1

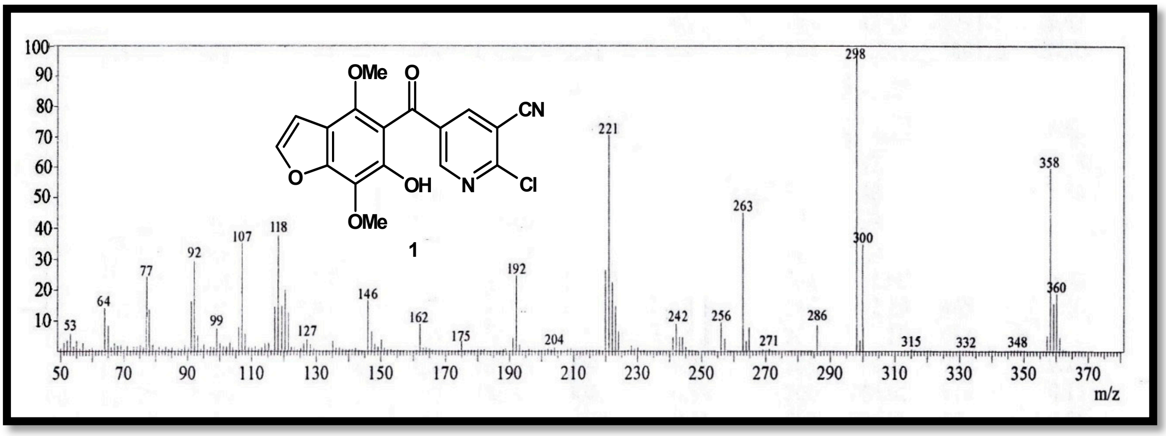


**Figure S3: Mass spectrum of compound 1**

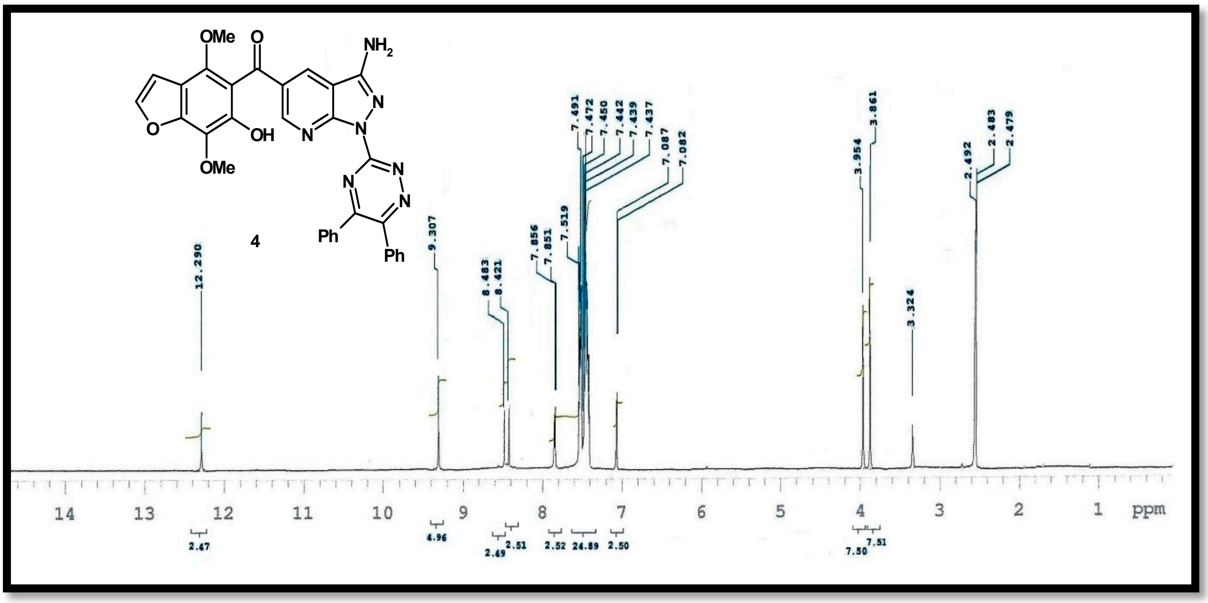


**Figure S4 (DMSO-$d_6$, 400 MHz): $^1$H NMR spectrum of compound 4**

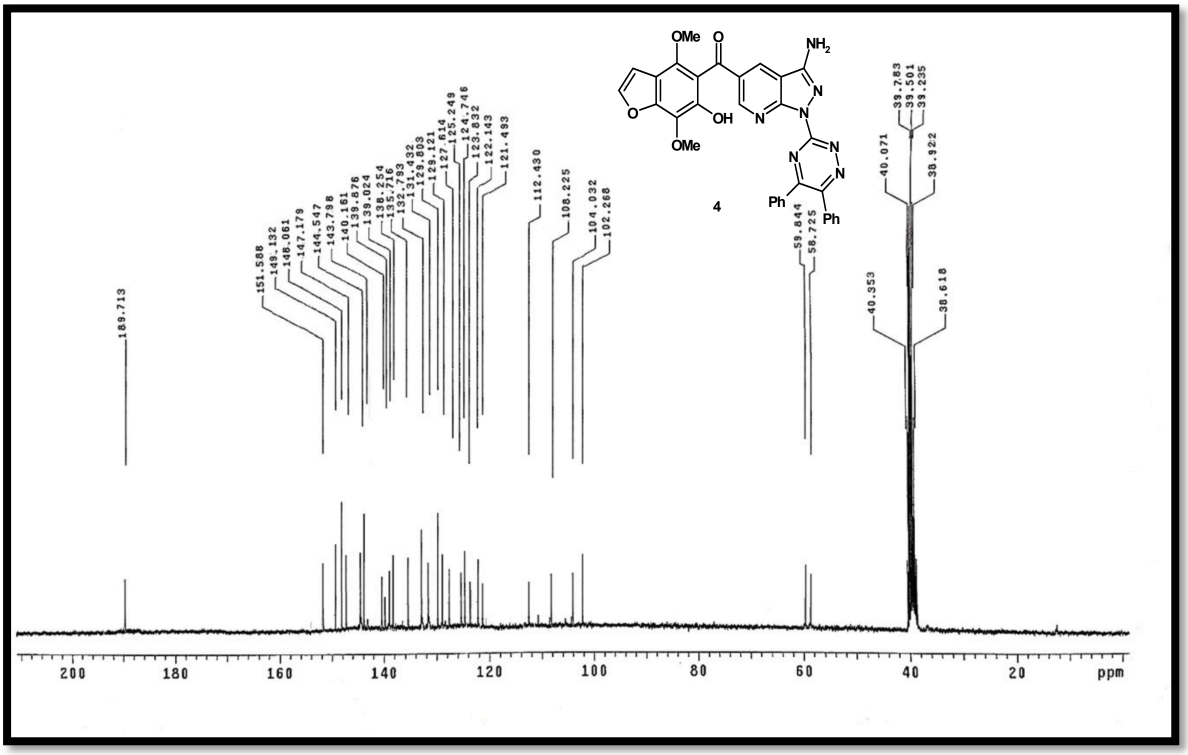


**Figure S5 (DMSO-$d_6$, 100 MHz): $^{13}$C NMR spectrum of compound 4**

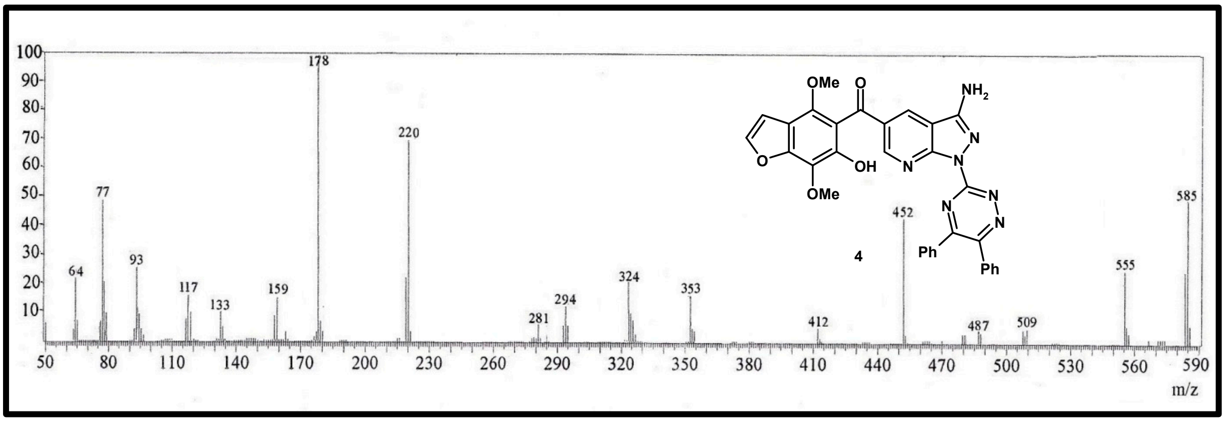


**Figure S6: Mass spectrum of compound 4**

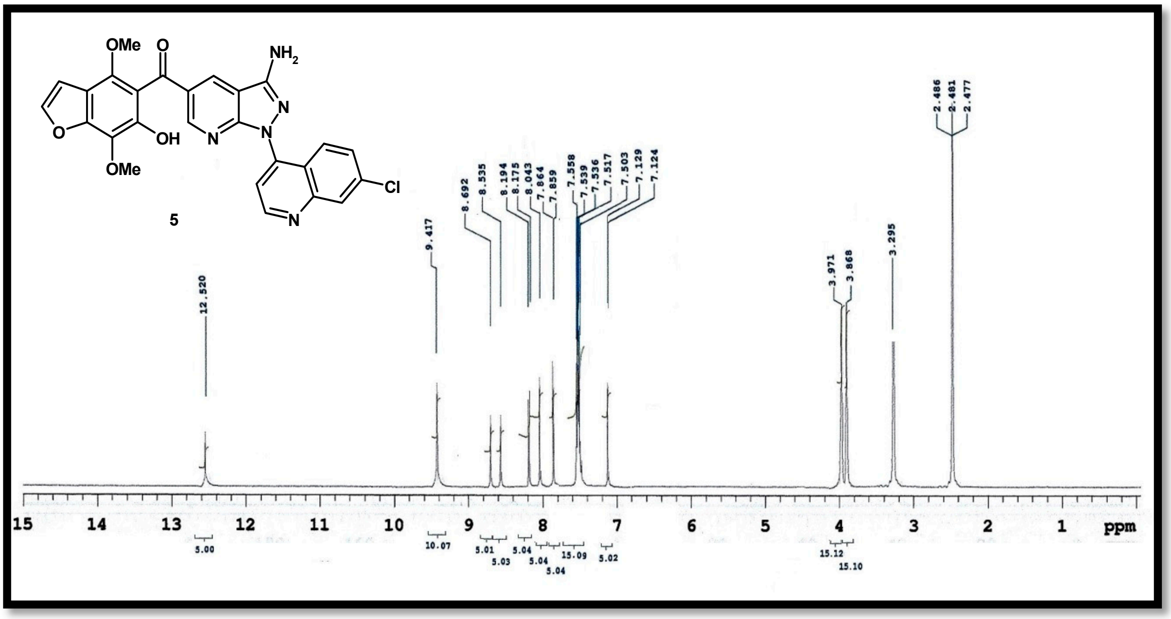


**Figure S7 (DMSO-$d_6$, 400 MHz): $^1$H NMR spectrum of compound 5**

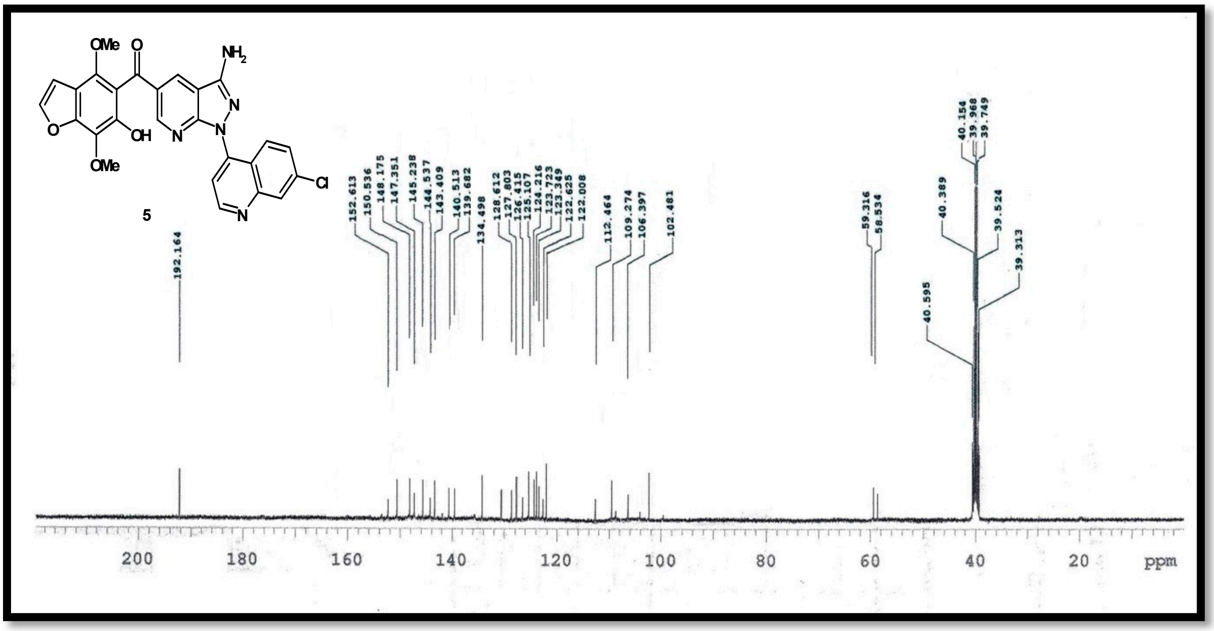


**Figure S8 (DMSO-$d_6$, 100 MHz): $^{13}$C NMR spectrum of compound 5**

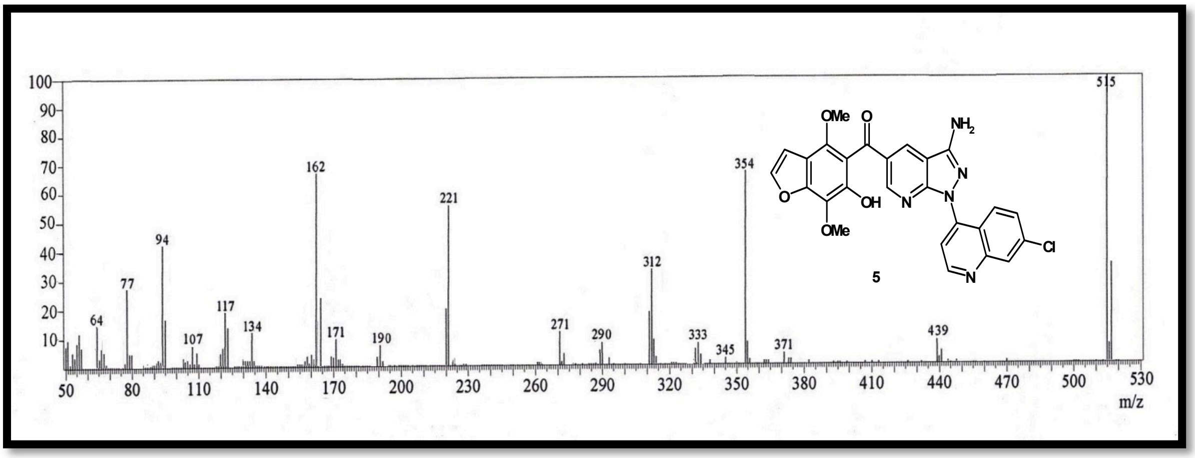


**Figure S9: Mass spectrum of compound 5**

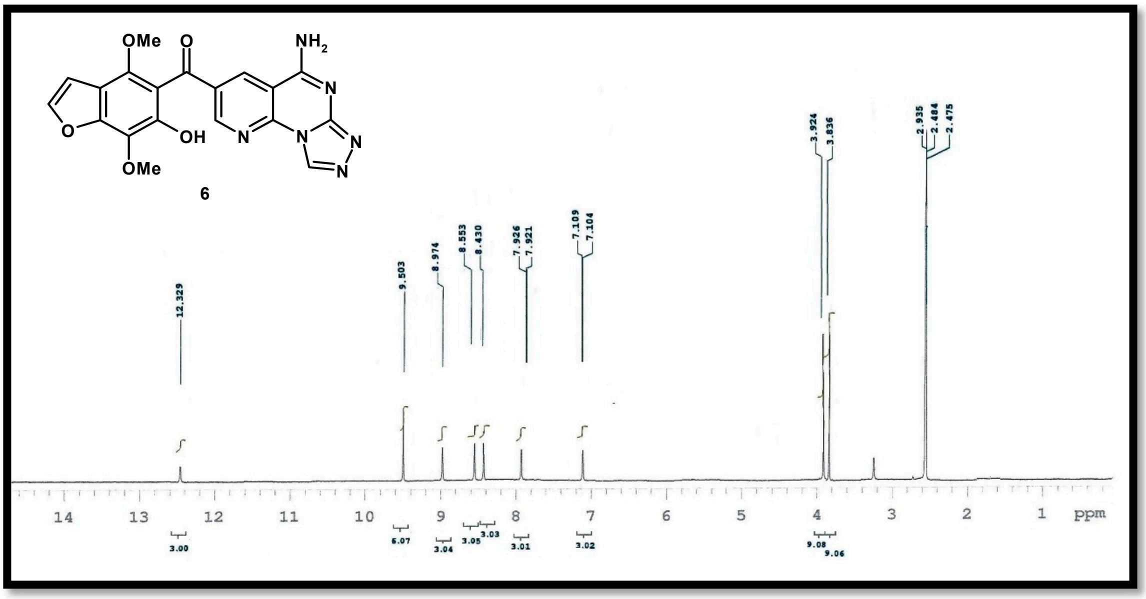


**Figure S10 (DMSO-$d_6$, 400 MHz): $^1$H NMR spectrum of compound 6**

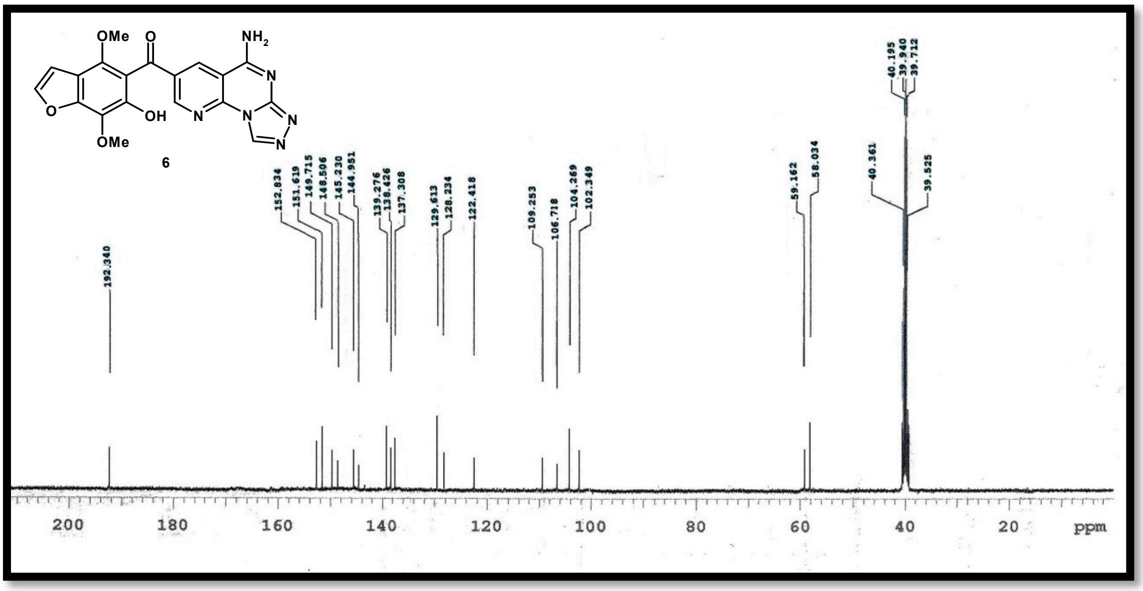

Figure S11 (DMSO-$d_6$, 100 MHz): $^{13}C$ NMR spectrum of compound 6

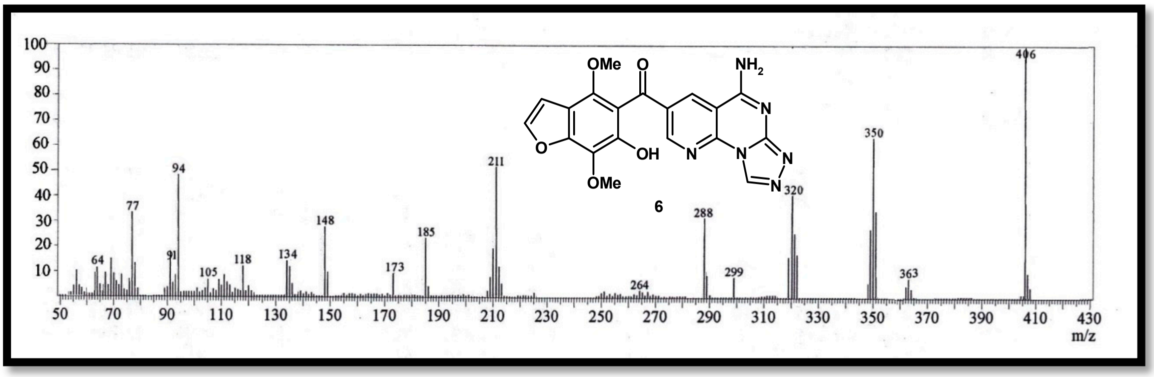

Figure S12: Mass spectrum of compound 6

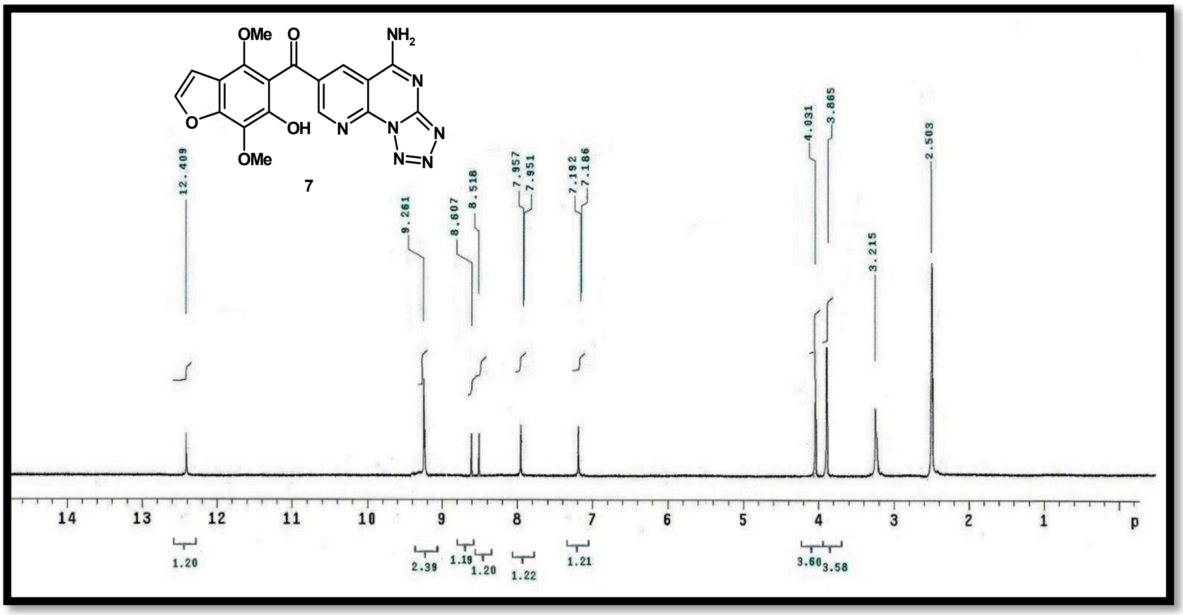


**Figure S13 (DMSO-$d_6$, 400 MHz): $^1$H NMR spectrum of compound 7**

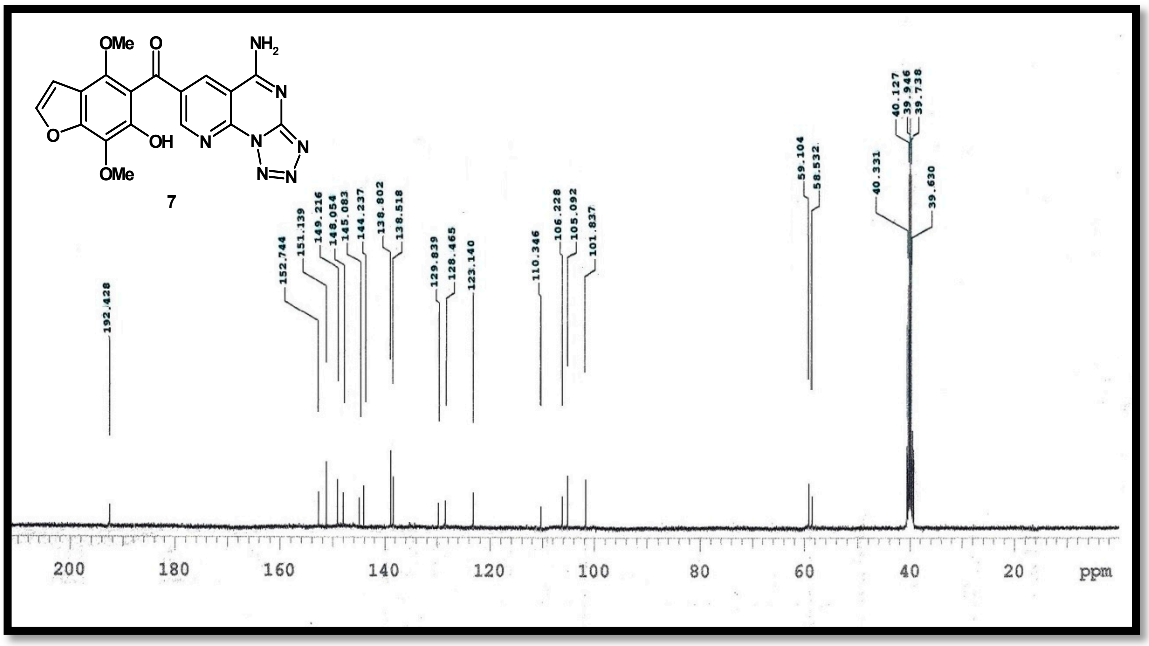


**Figure S14 (DMSO-$d_6$, 100 MHz): $^{13}$C NMR spectrum of compound 7**

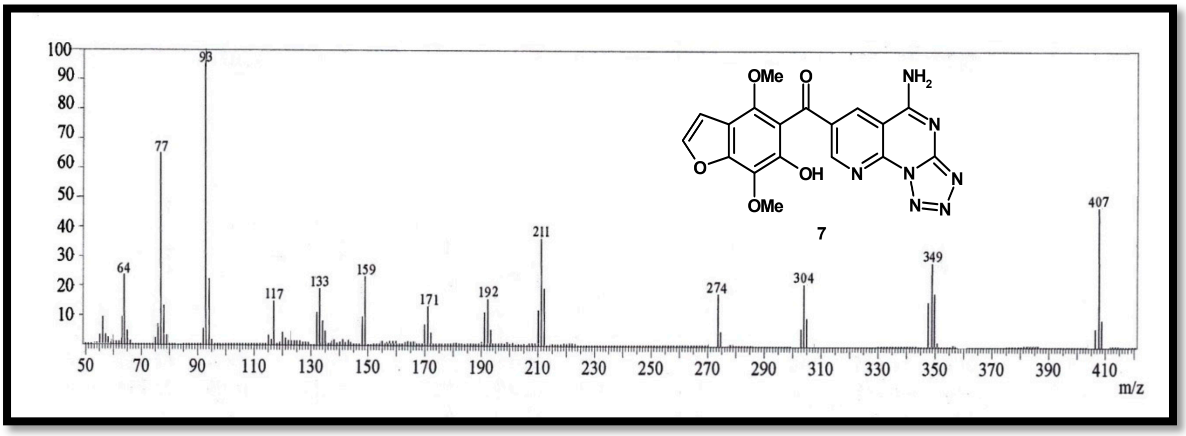

**Figure S15: Mass spectrum of compound 7**

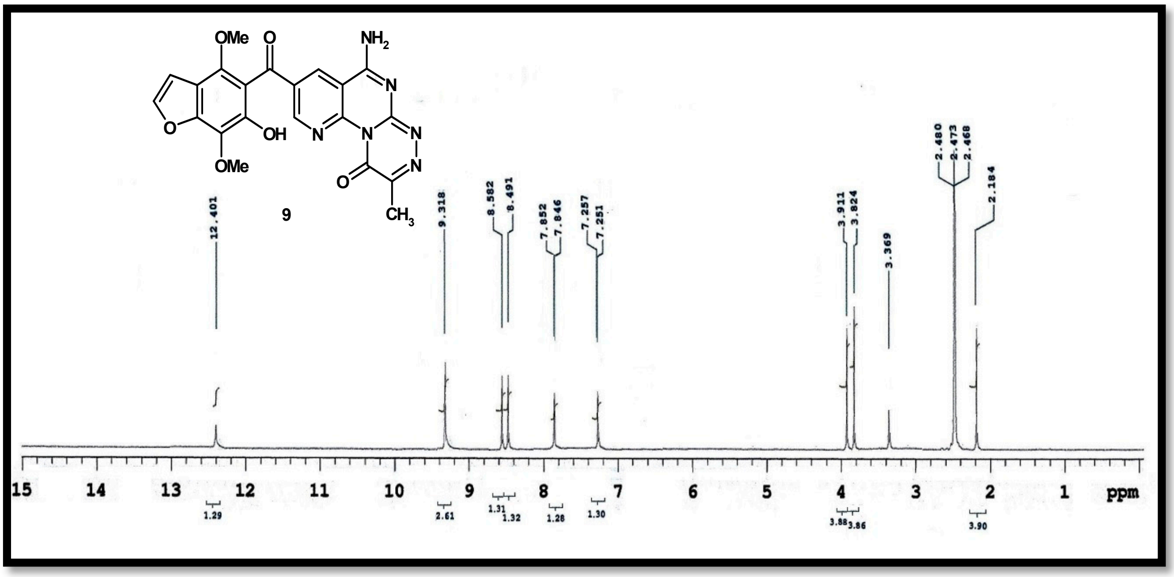

**Figure S16 (DMSO-$d_6$, 400 MHz): $^1$H NMR spectrum of compound 9**

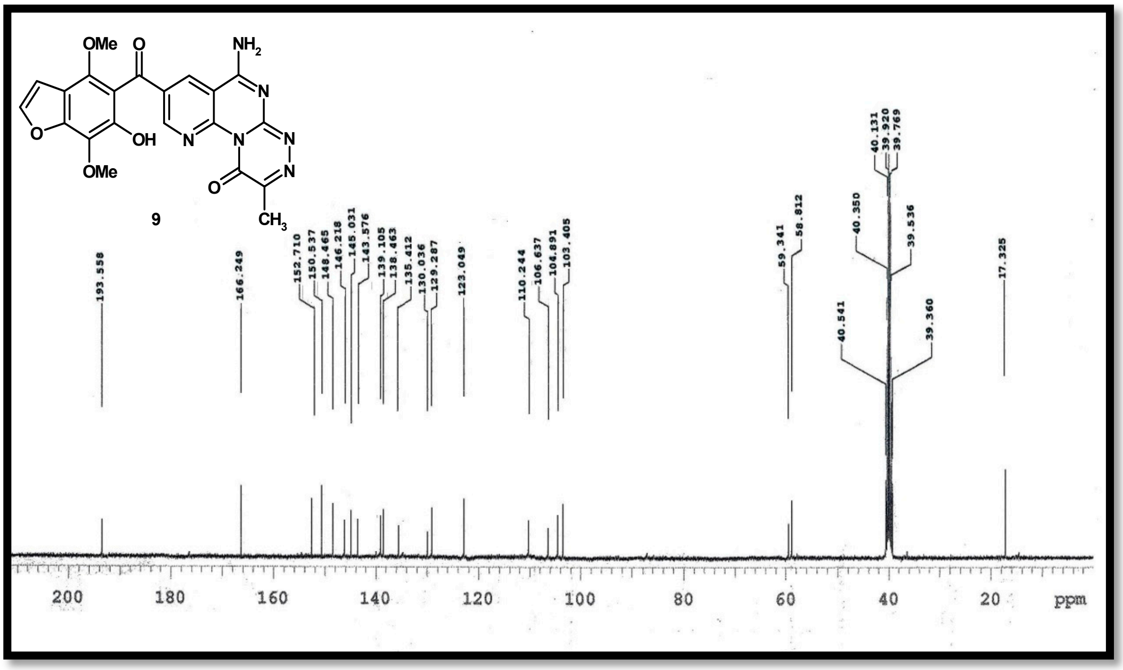

**Figure S17 (DMSO-$d_6$, 100 MHz): $^{13}$C NMR spectrum of compound 9**

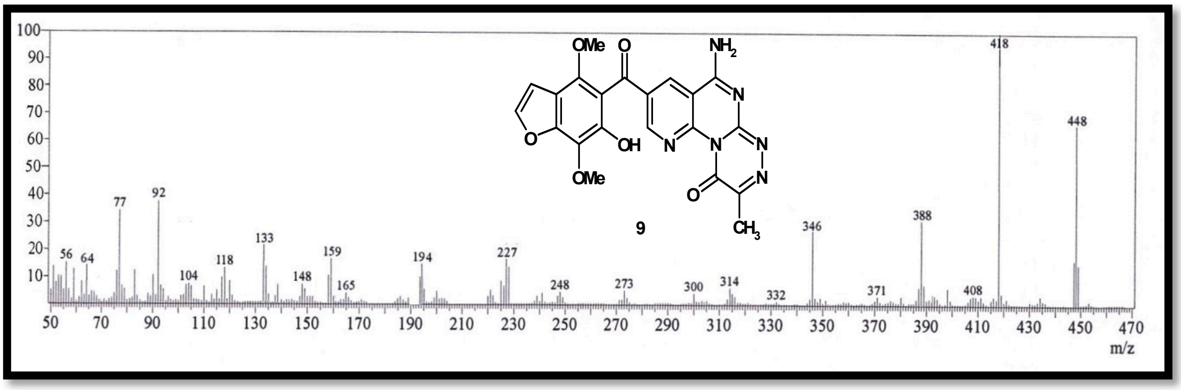

**Figure S18: Mass spectrum of compound 9**

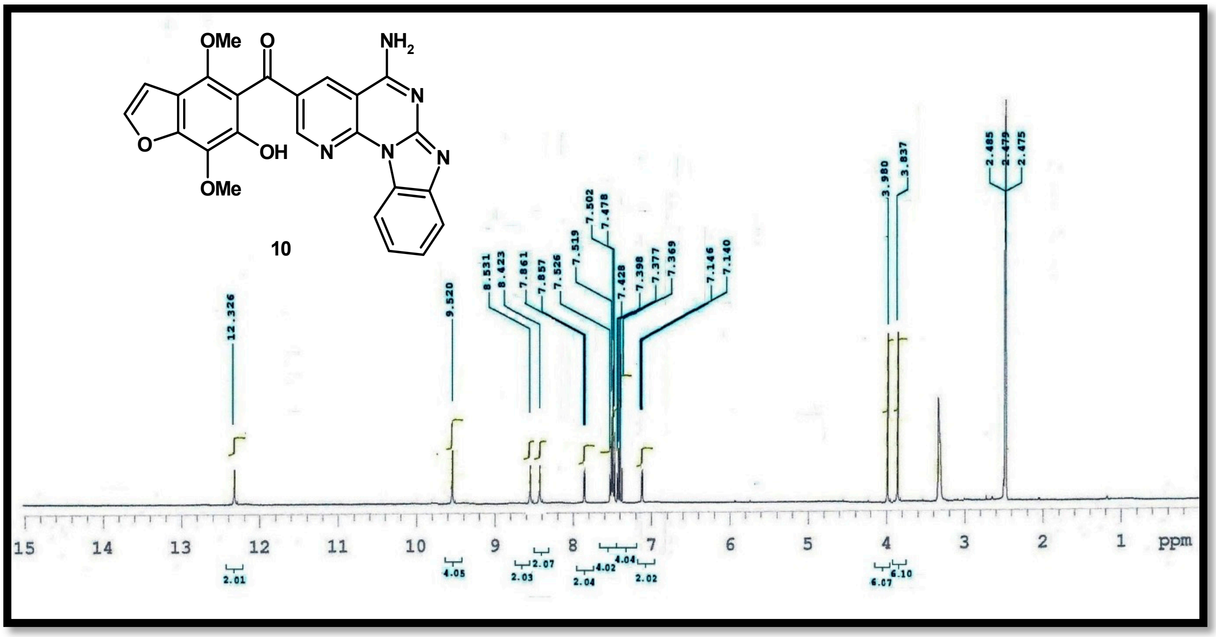


**Figure S19 (DMSO-$d_6$, 400 MHz): $^1$H NMR spectrum of compound 10**

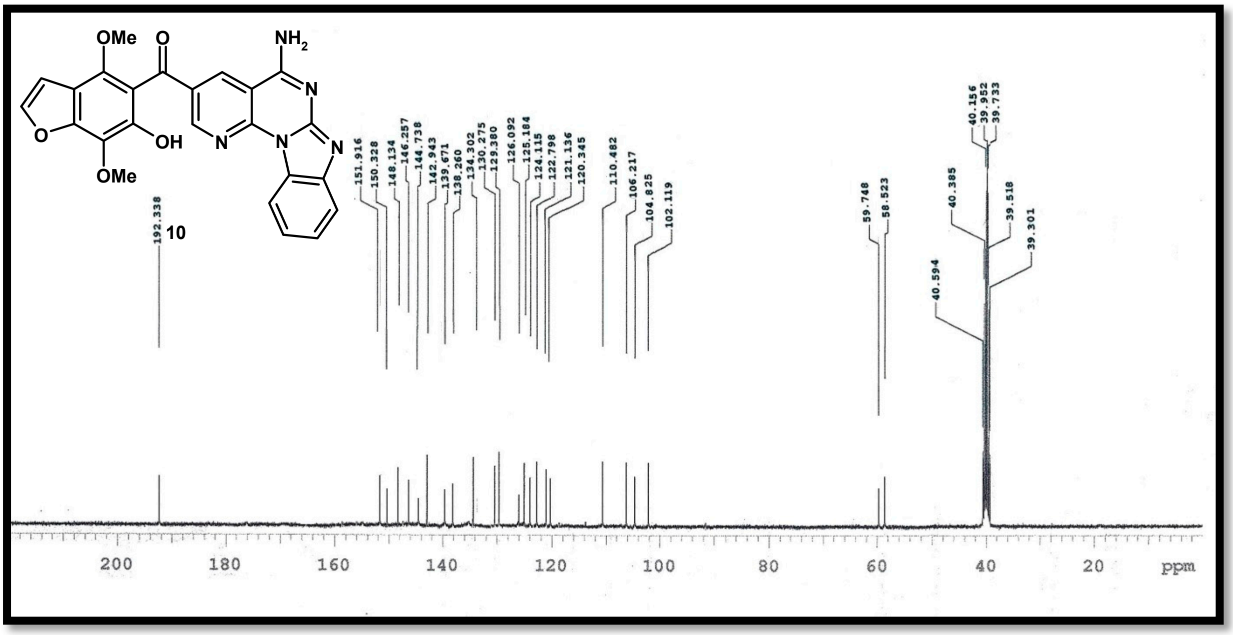


**Figure S20 (DMSO-$d_6$, 100 MHz): $^{13}$C NMR spectrum of compound 10**

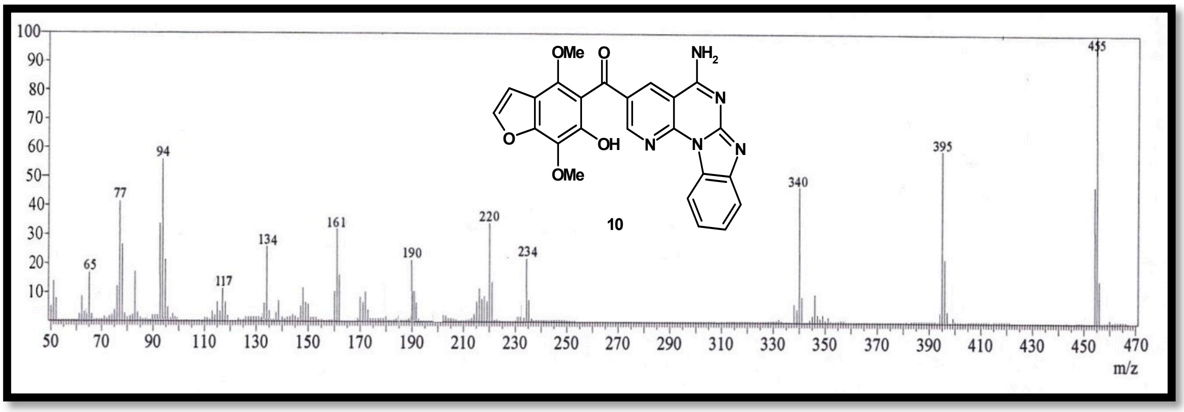


**Figure S21: Mass spectrum of compound 10**

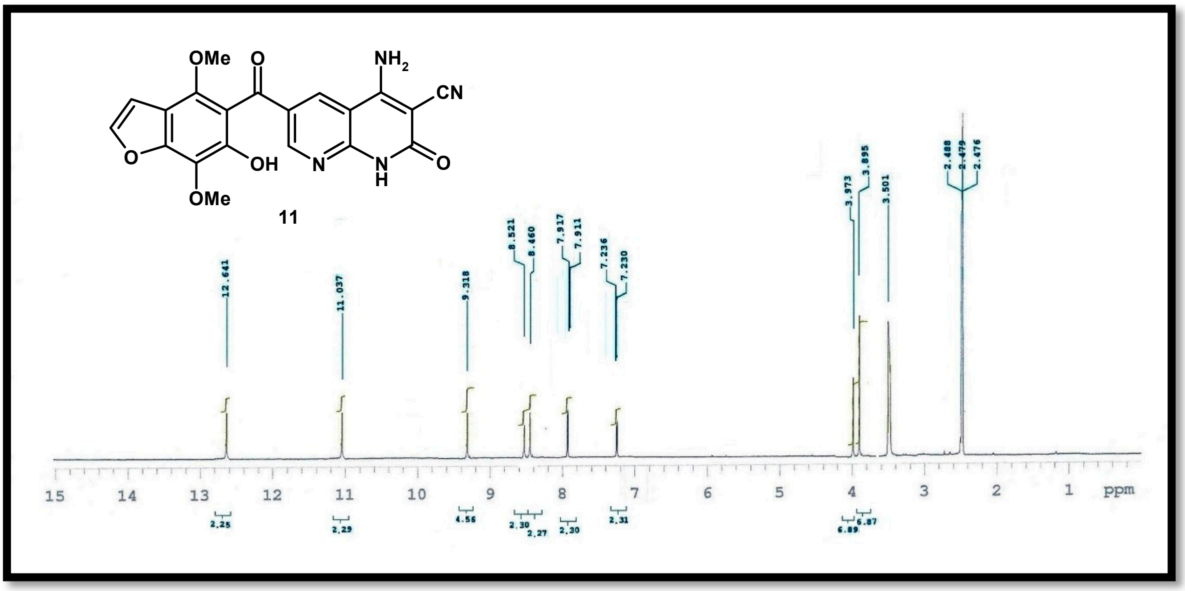


**Figure S22 (DMSO-$d_6$, 400 MHz): $^1$H NMR spectrum of compound 11**

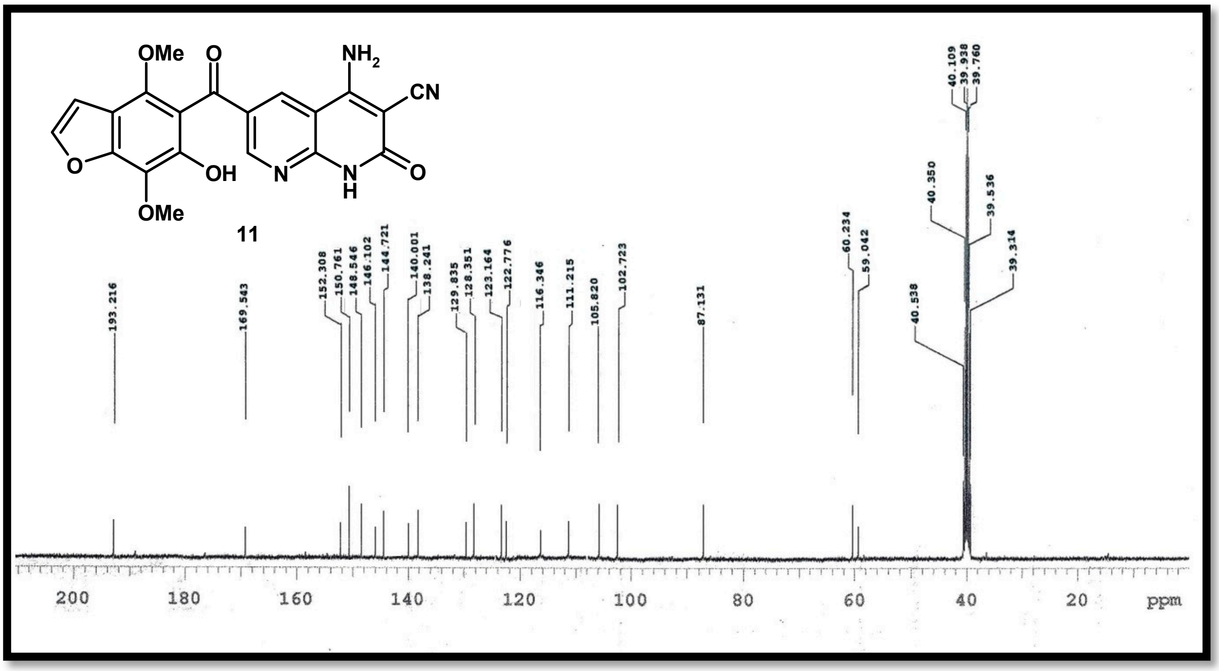

**Figure S23 (DMSO-$d_6$, 400 MHz): $^1$H NMR spectrum of compound 11**

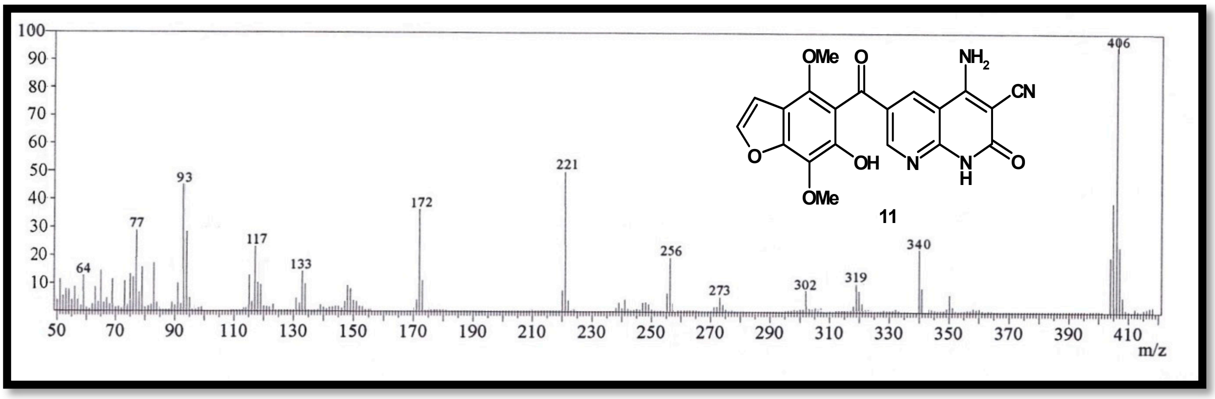

**Figure S24: Mass spectrum of compound 11**

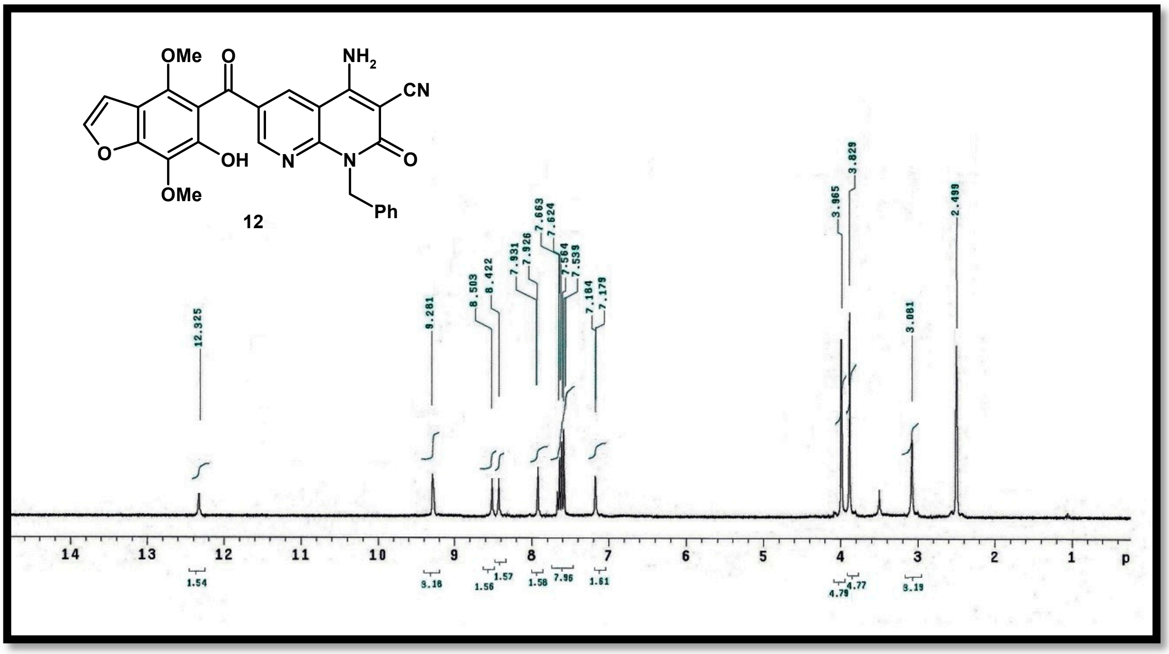


**Figure S25 (DMSO-$d_6$, 400 MHz): $^1$H NMR spectrum of compound 12**

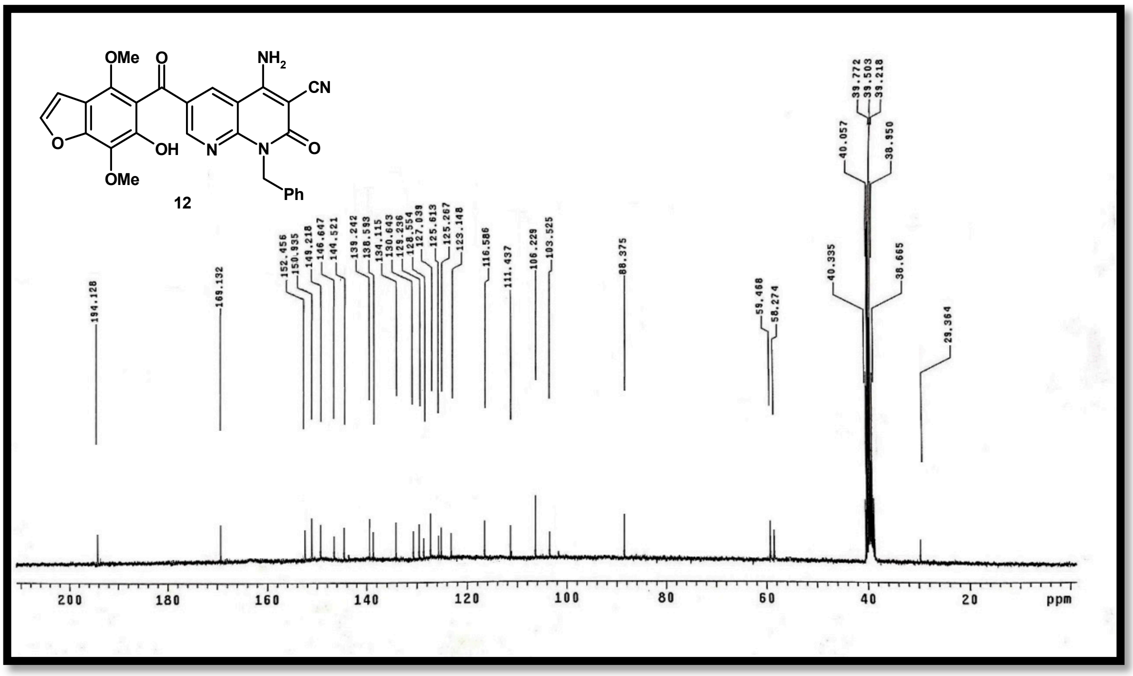


**Figure S26 (DMSO-$d_6$, 100 MHz): $^{13}$C NMR spectrum of compound 12**

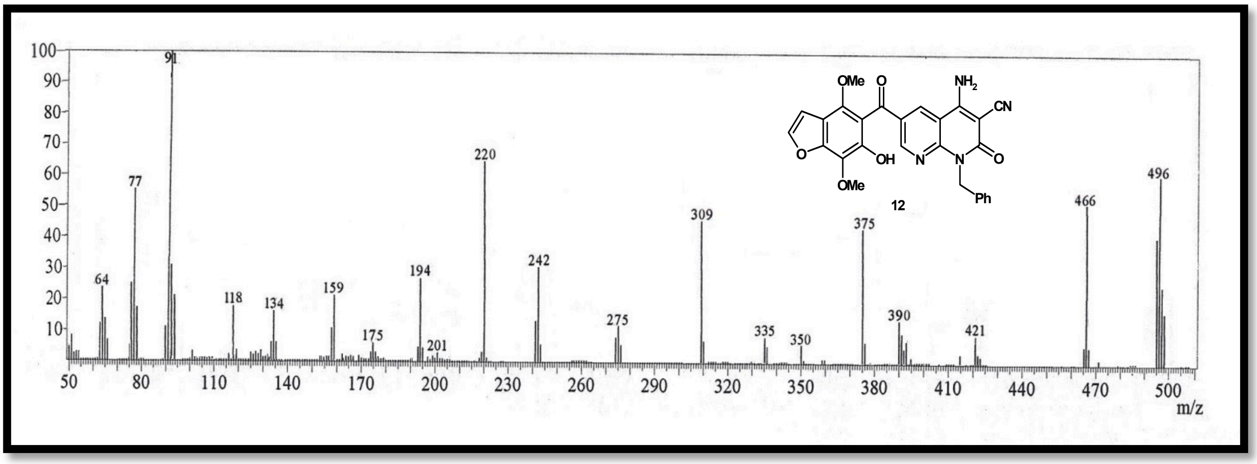


**Figure S27: Mass spectrum of compound 12**

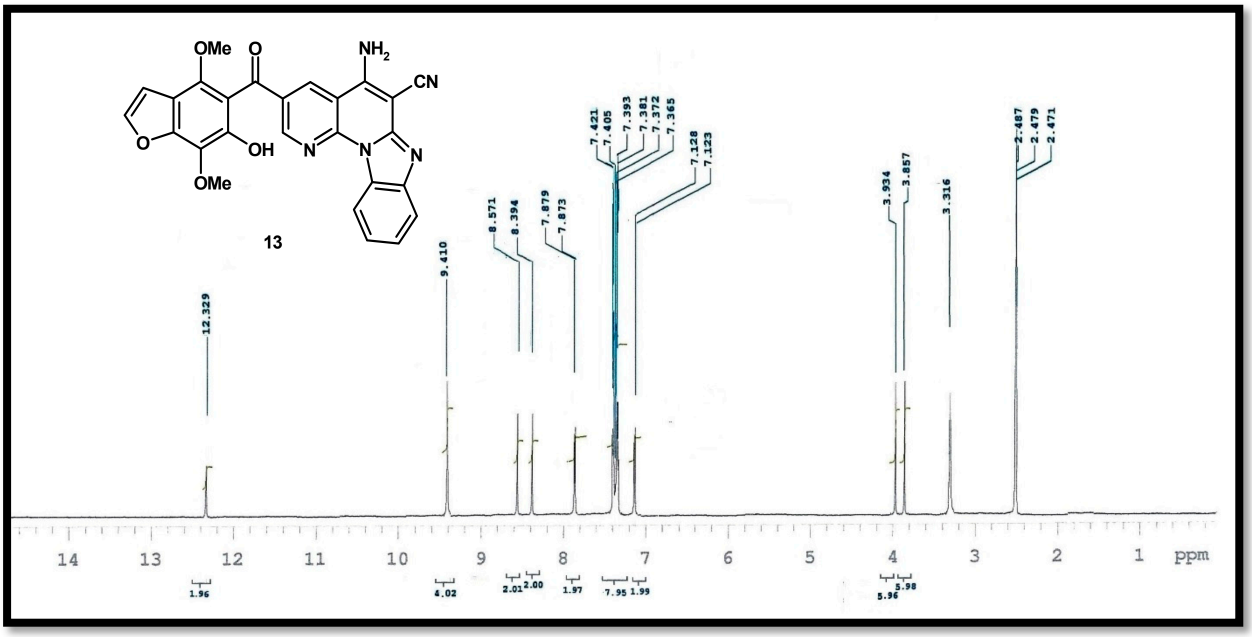


**Figure S28 (DMSO-$d_6$, 400 MHz): $^1$H NMR spectrum of compound 13**

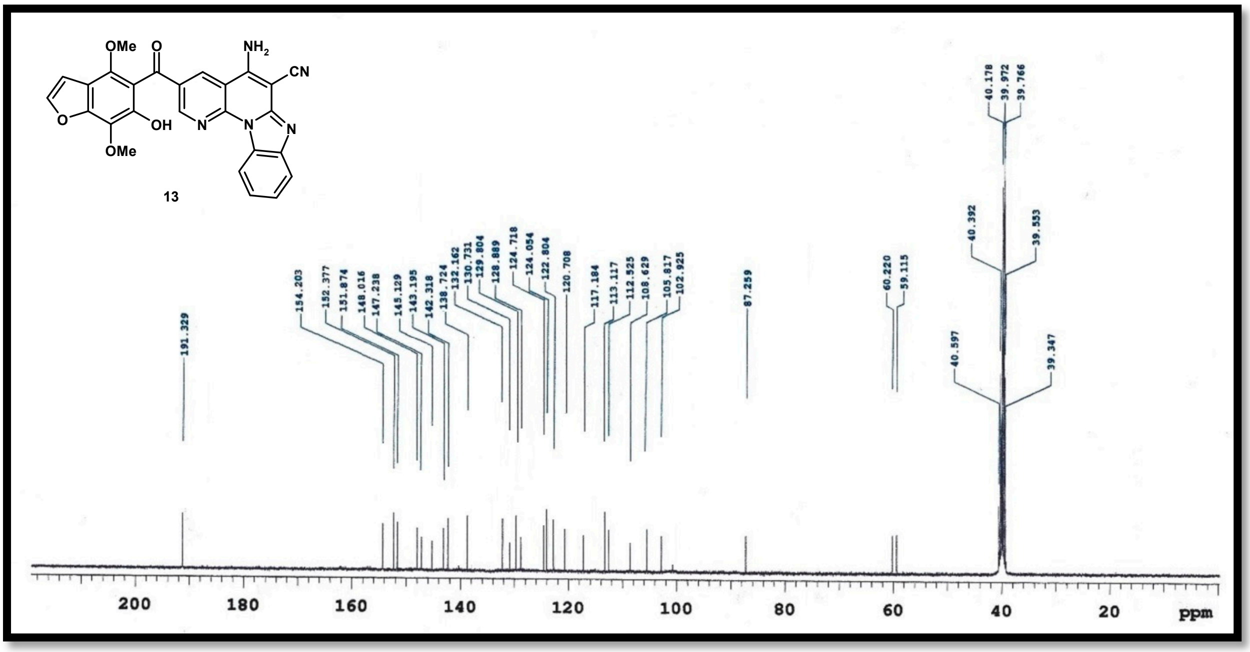


**Figure S29 (DMSO-$d_6$, 100 MHz): $^{13}$C NMR spectrum of compound 13**

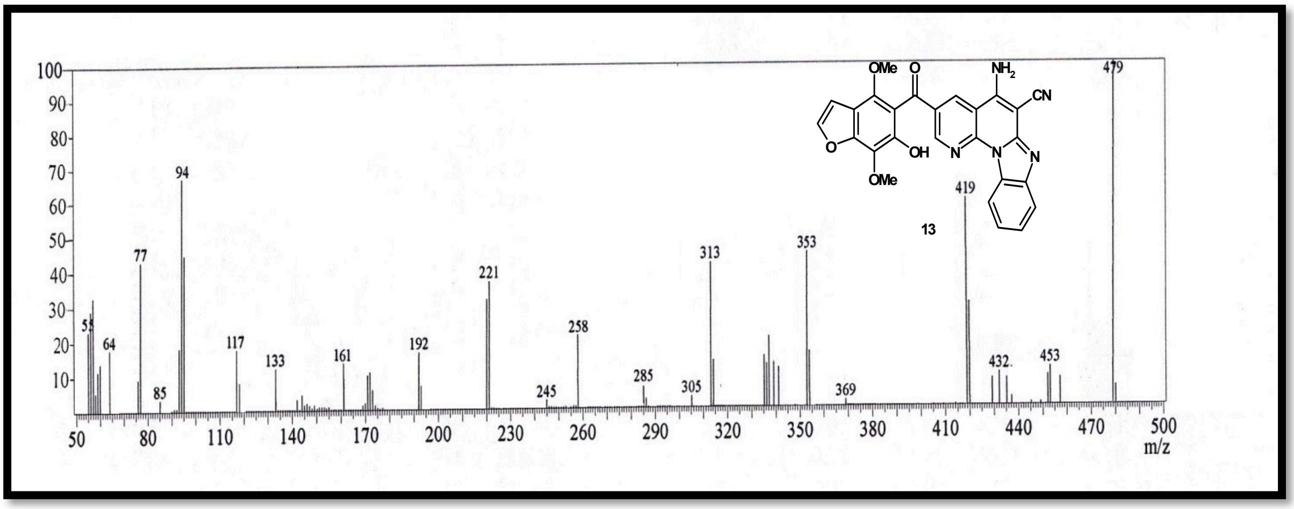


**Figure S30: Mass spectrum of compound 13**

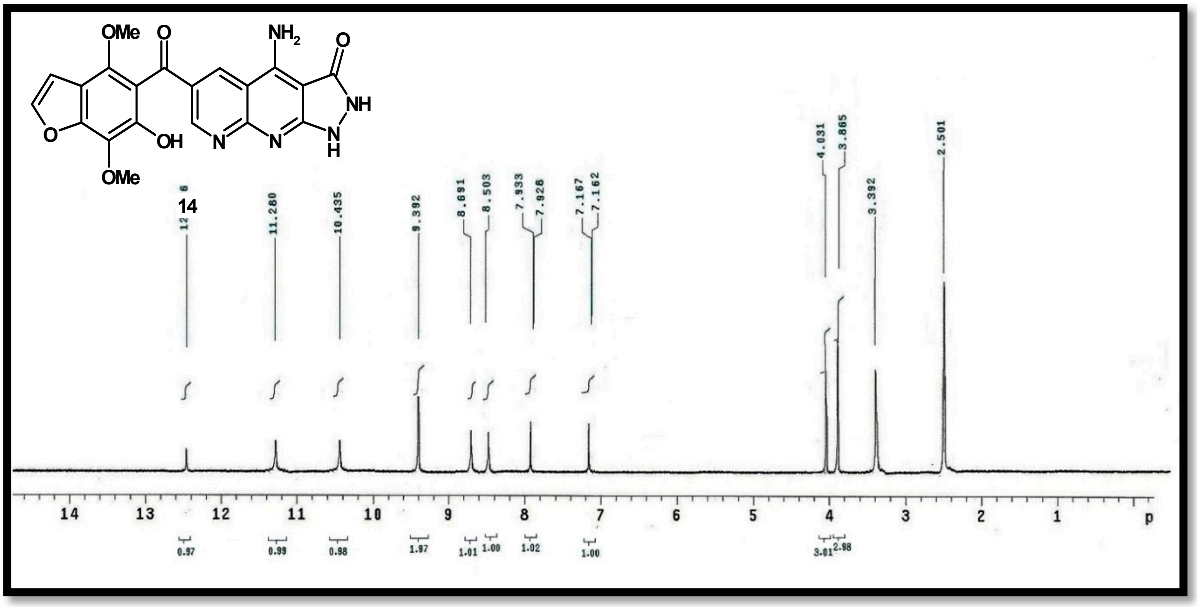


**Figure S31 (DMSO-$d_6$, 400 MHz): $^1$H NMR spectrum of compound 14**

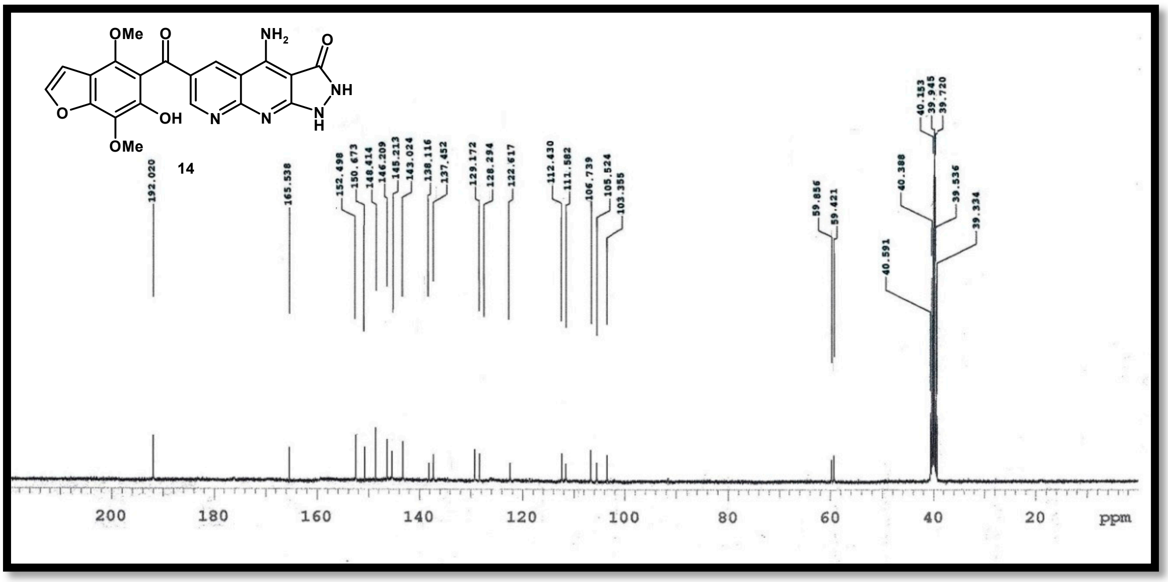


**Figure S32 (DMSO-$d_6$, 100 MHz): $^{13}$C NMR spectrum of compound 14**

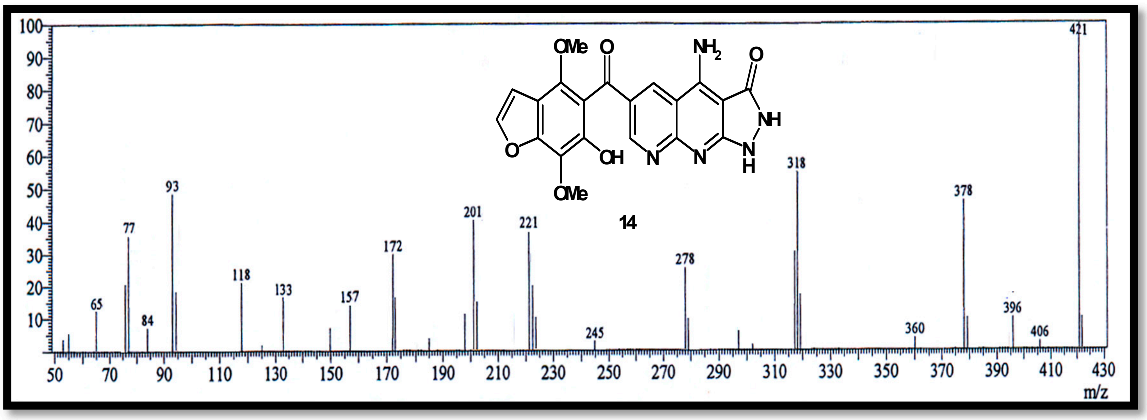


**Figure S33: Mass spectrum of compound 14**

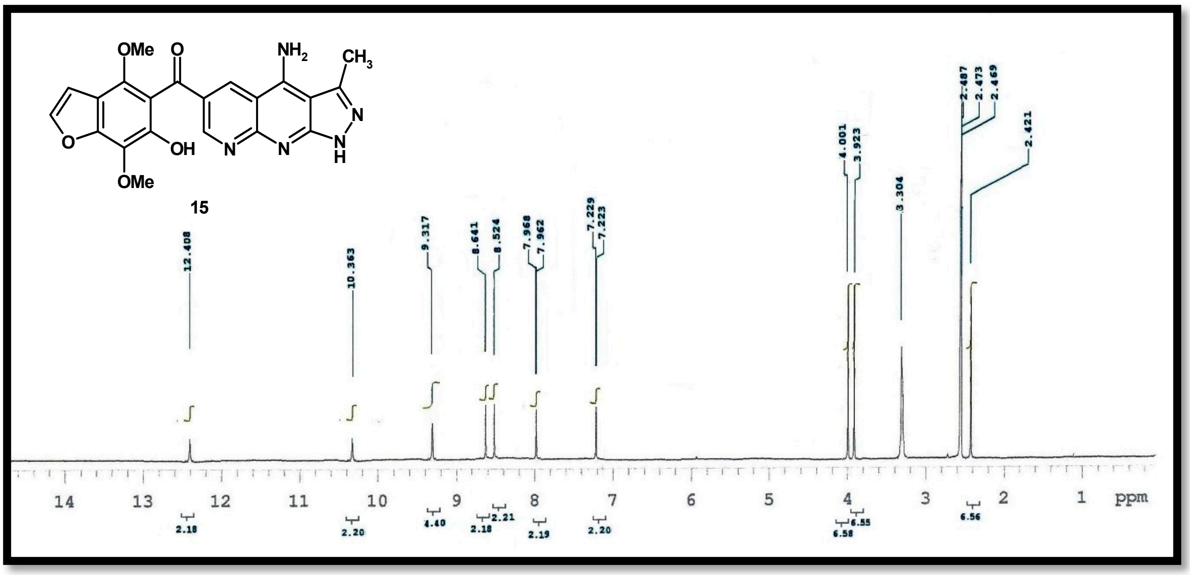


**Figure S34 (DMSO-$d_6$, 400 MHz): $^1$H NMR spectrum of compound 15**

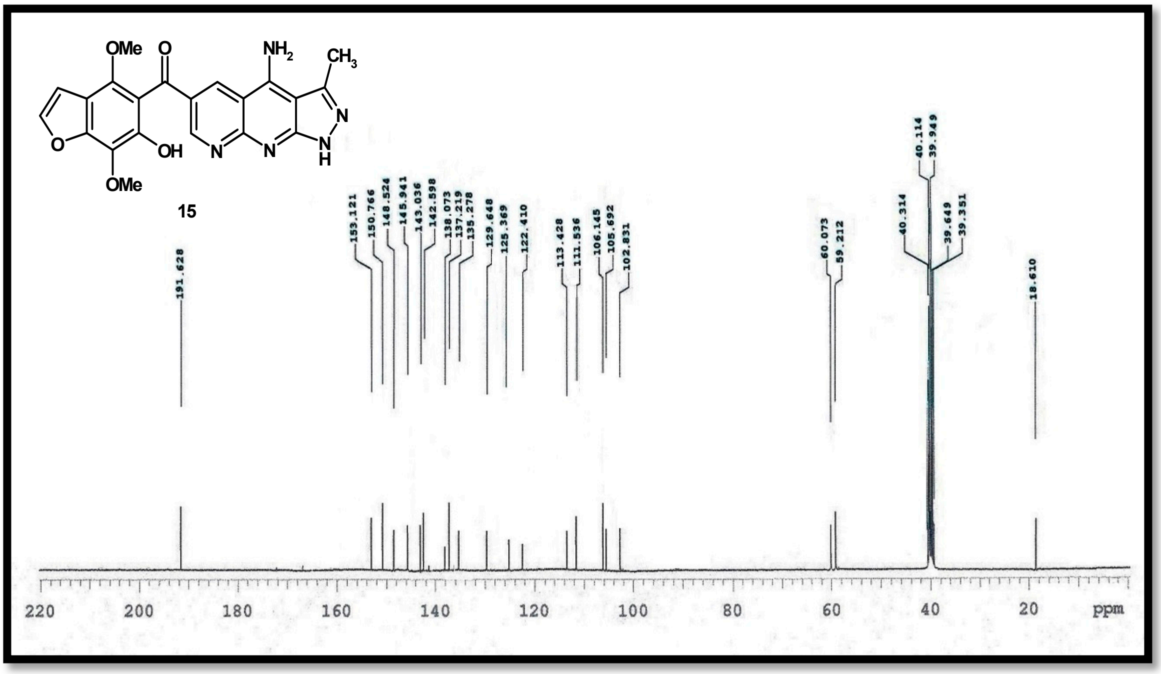


**Figure S35 (DMSO-$d_6$, 100 MHz): $^{13}$C NMR spectrum of compound 15**

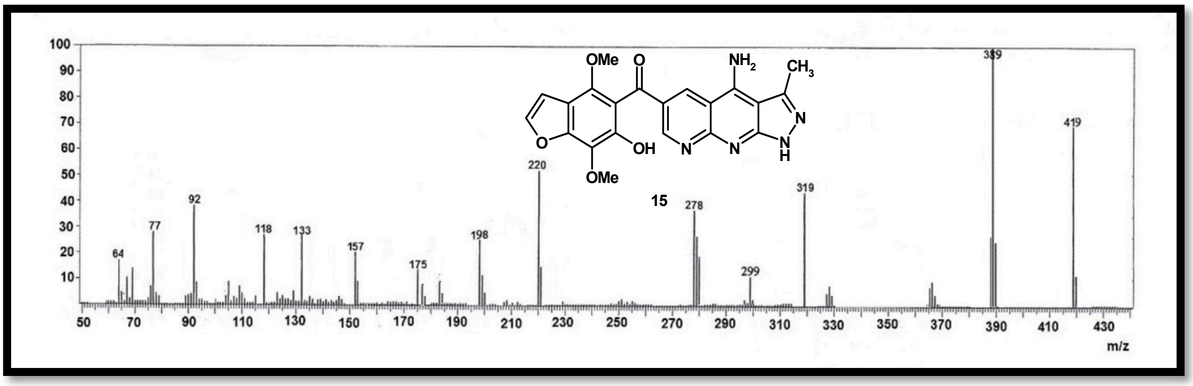


**Figure S36: Mass spectrum of compound 15**

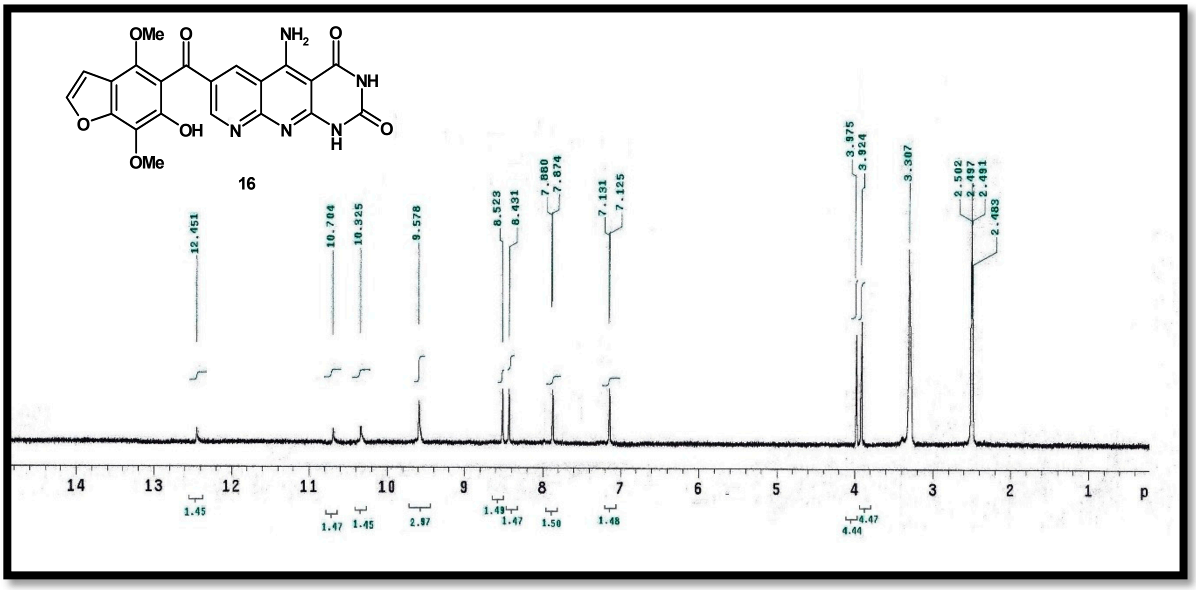

**Figure S37 (DMSO-$d_6$, 400 MHz): $^1$H NMR spectrum of compound 16**

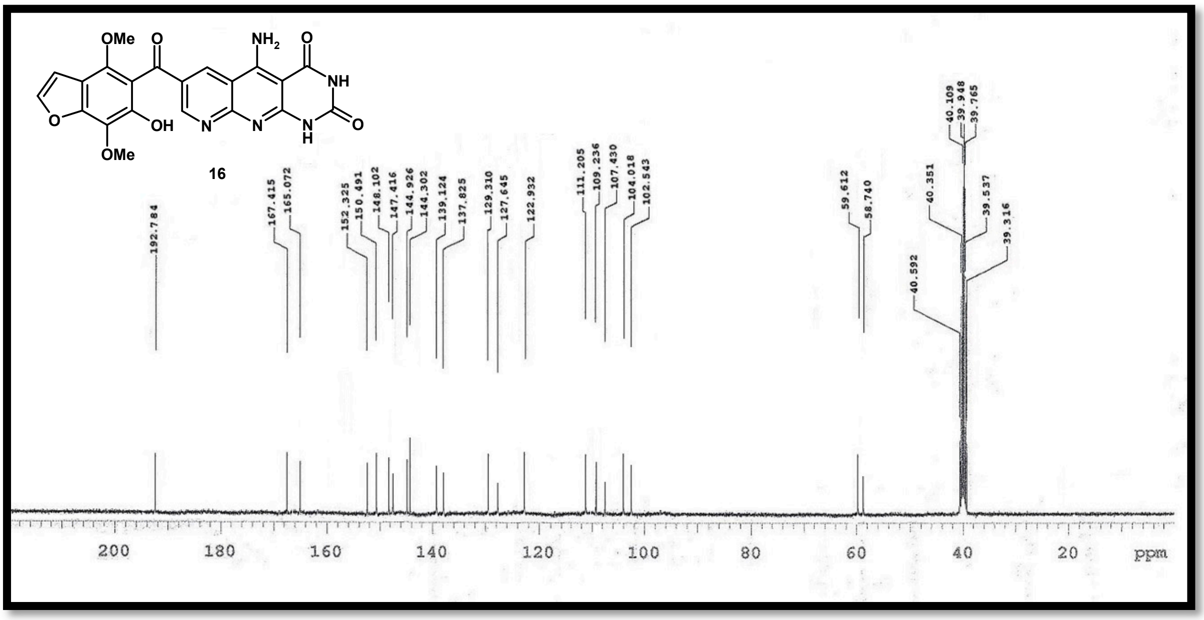

**Figure S38 (DMSO-$d_6$, 100 MHz): $^{13}$C NMR spectrum of compound 16**

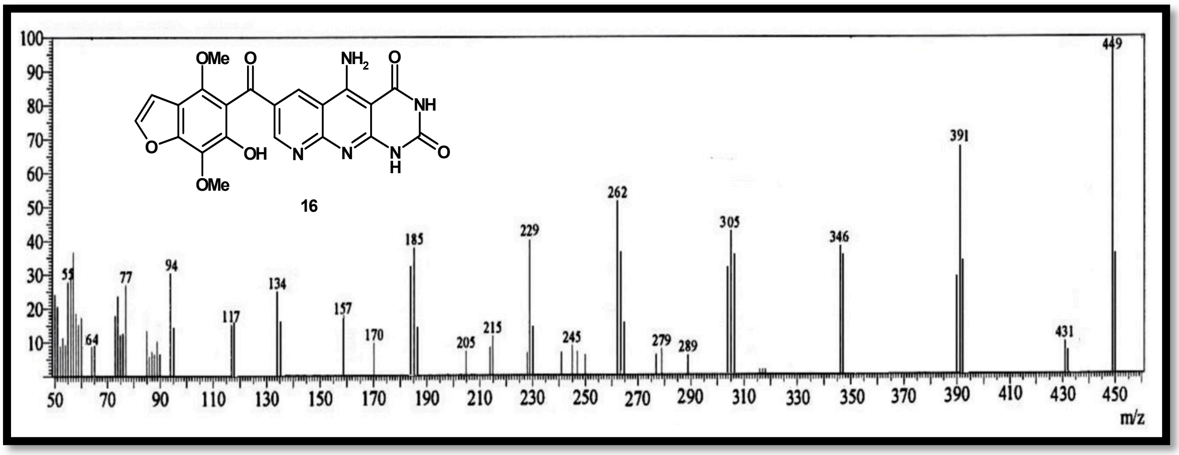


**Figure S39: Mass spectrum of compound 16**

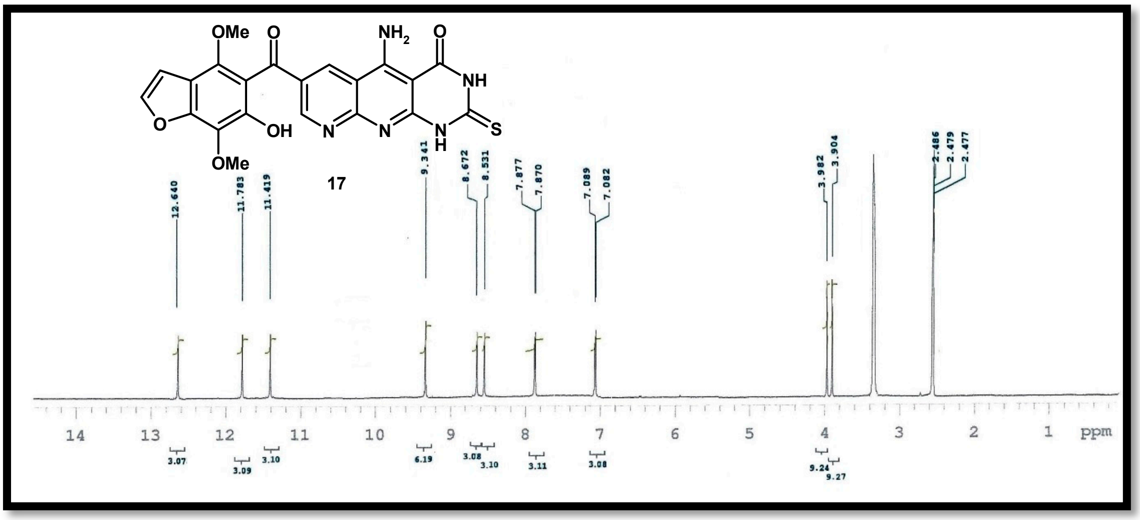


**Figure S40 (DMSO-$d_6$, 400 MHz): $^1$H NMR spectrum of compound 17**

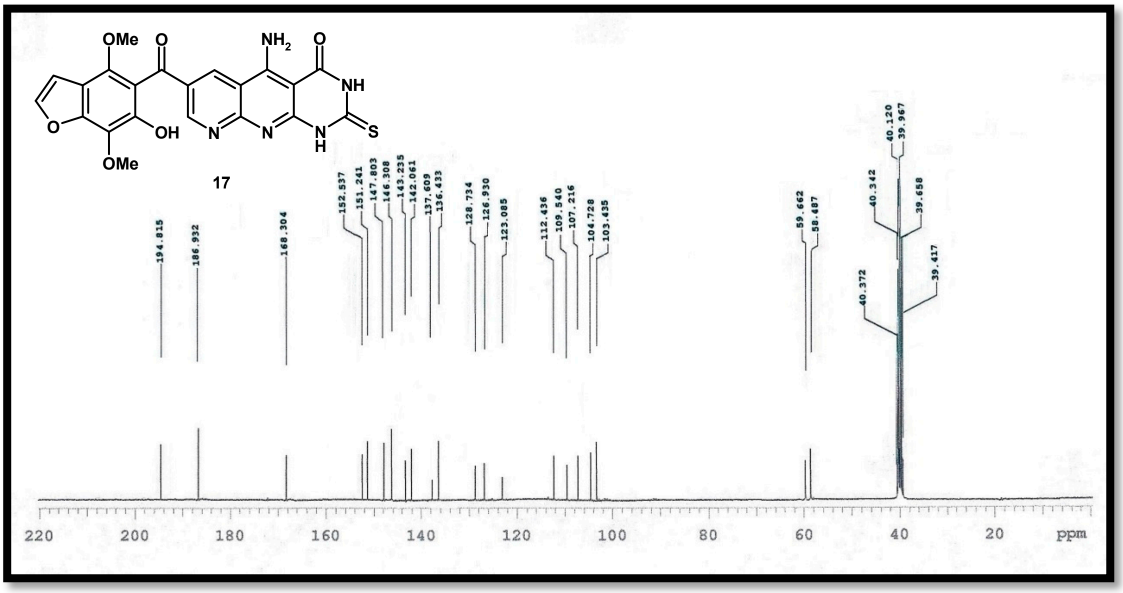

**Figure S41 (DMSO-$d_6$, 100 MHz): $^{13}$C NMR spectrum of compound 17**

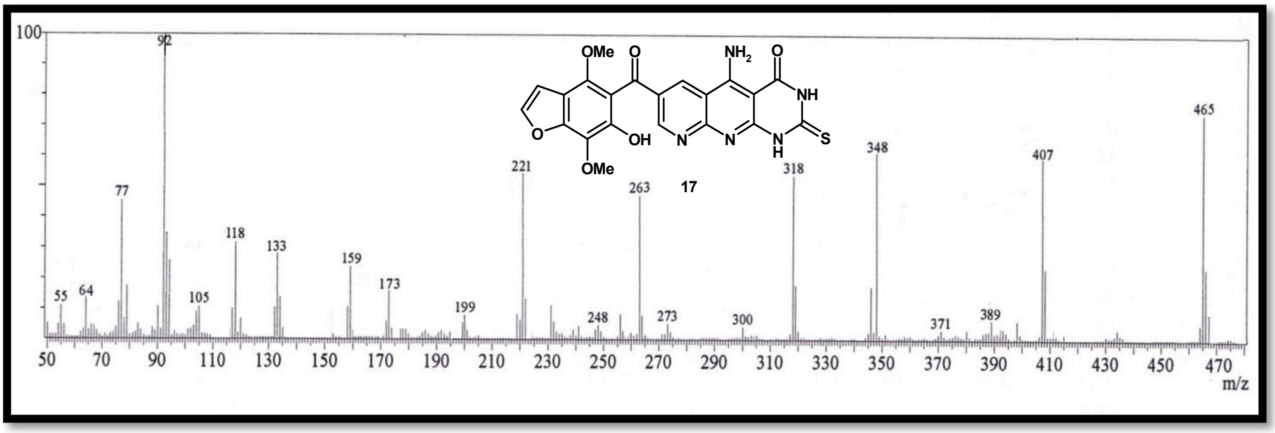

**Figure S42: Mass spectrum of compound 17**

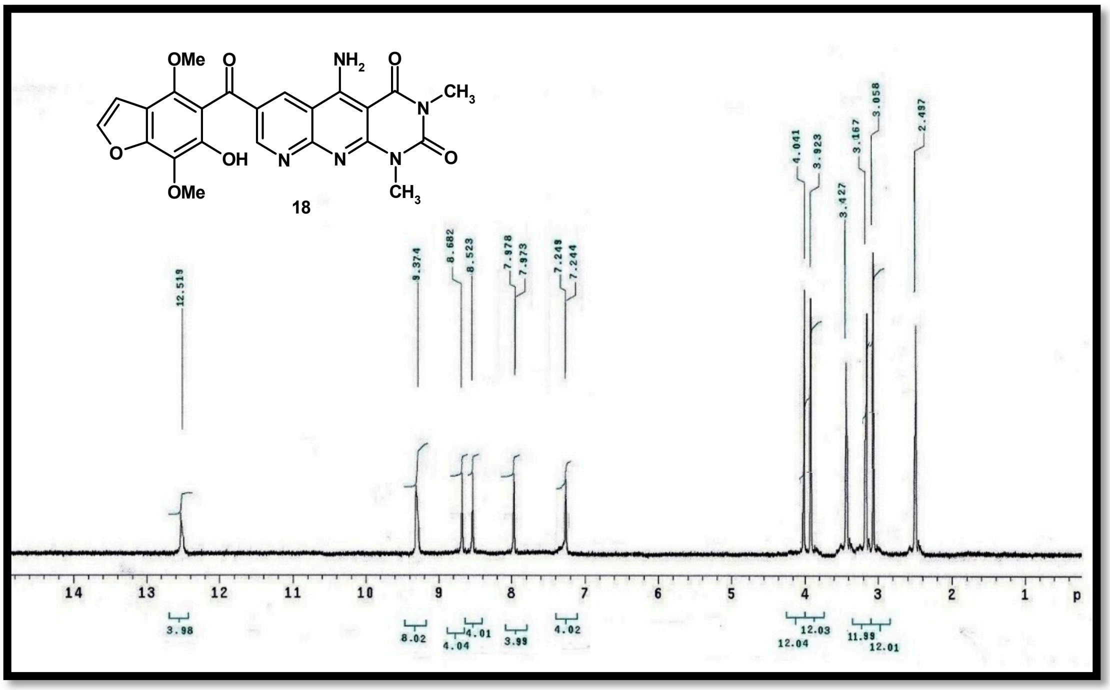

**Figure S43 (DMSO-$d_6$, 400 MHz): $^{1}$H NMR spectrum of compound 18**

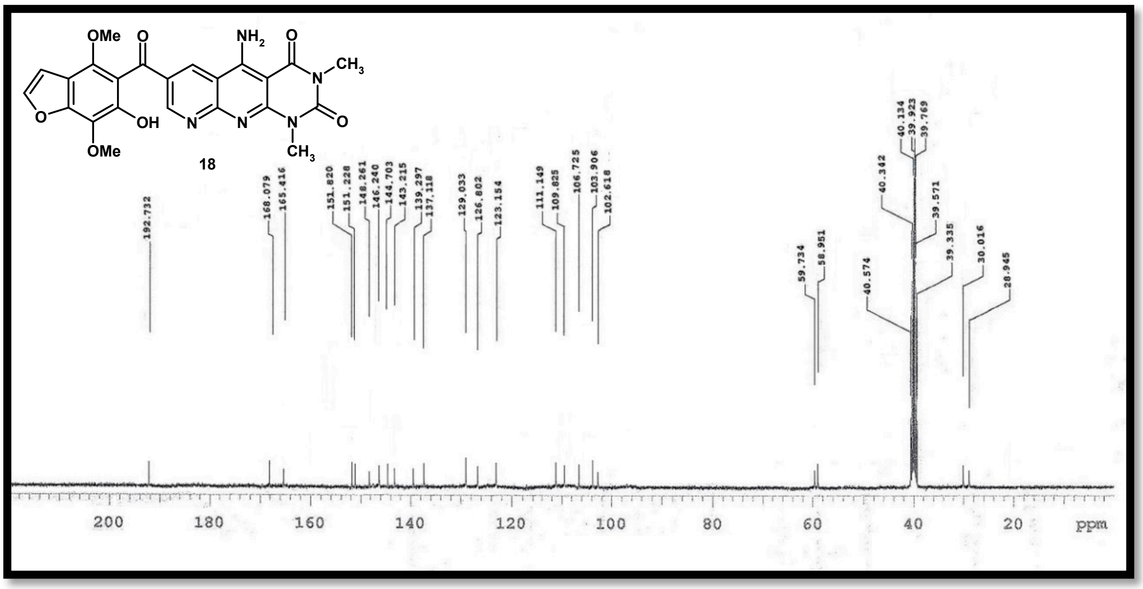

**Figure S44 (DMSO-$d_6$, 100 MHz): $^{13}$C NMR spectrum of compound 18**

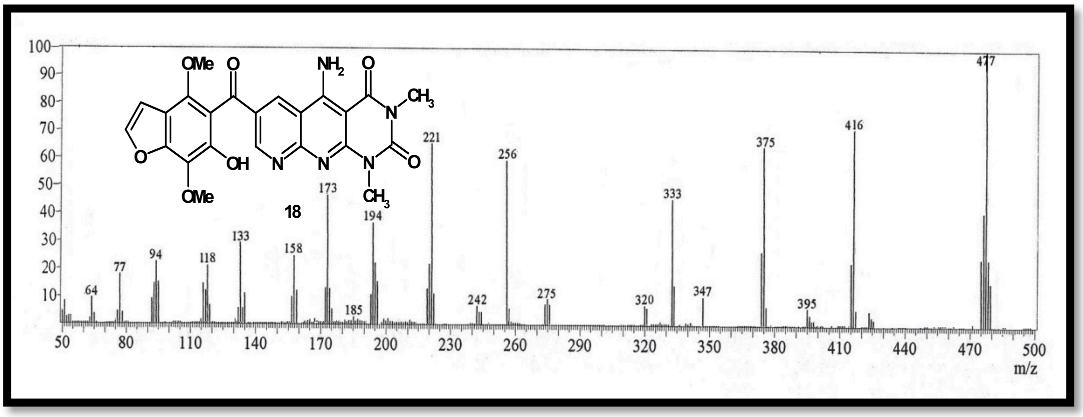


**Figure S45: Mass spectrum of compound 18**

## B) Antimicrobial efficiency of the synthesized compounds with respected to references drugs

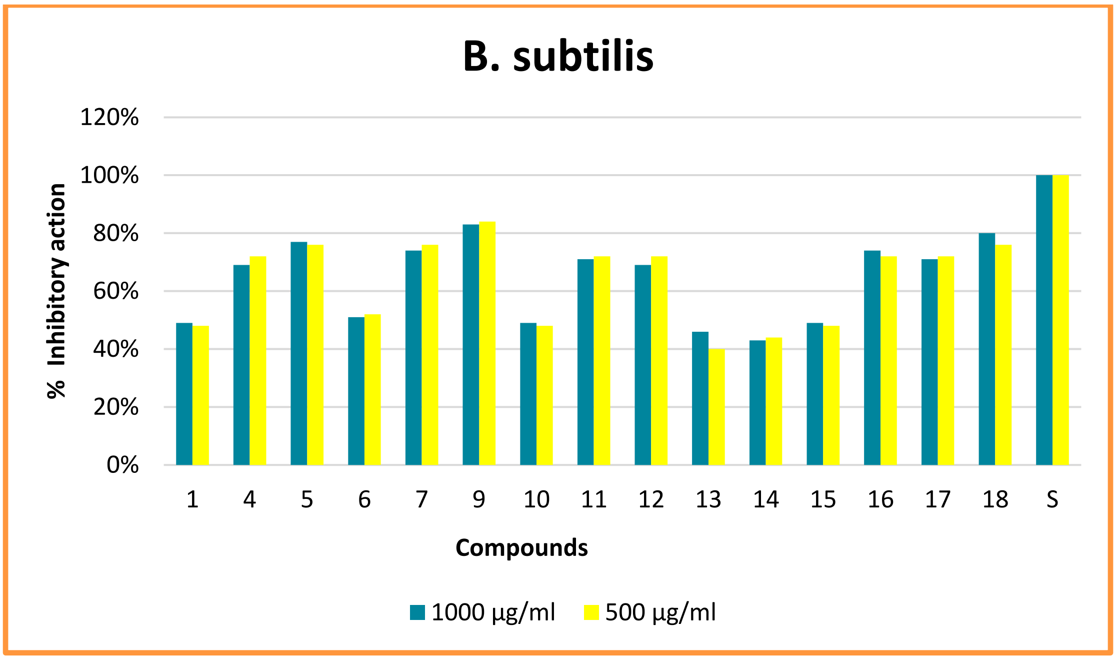


**Chart S1:** The antibacterial efficiency of synthesized compounds against *B. Subtilis*

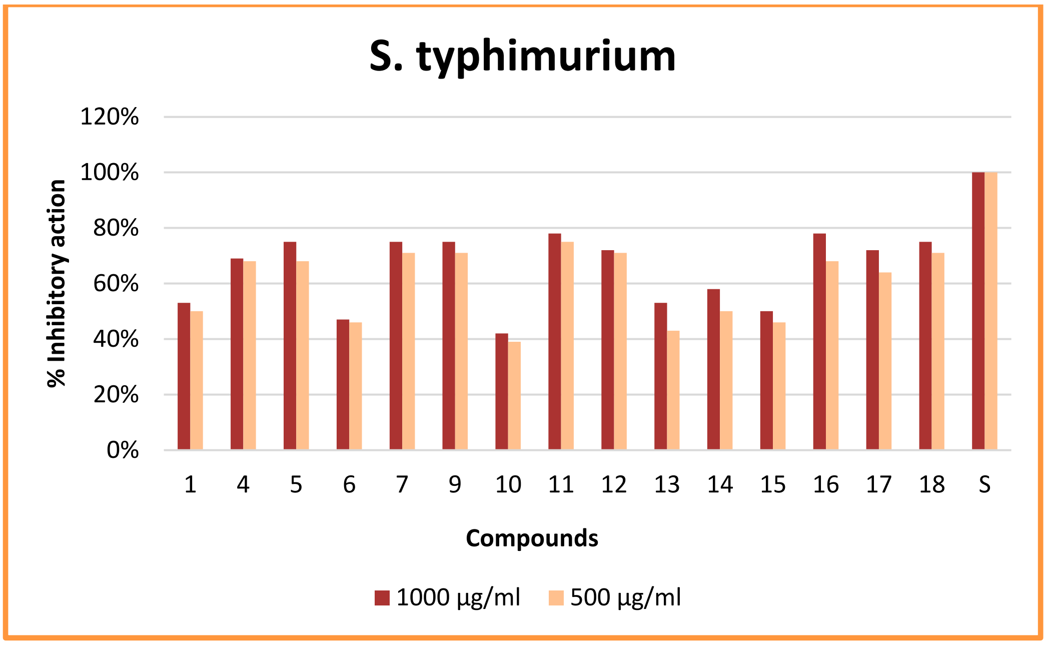


**Chart S2:** The antibacterial efficiency of synthesized compounds against *S. Typhimurium*

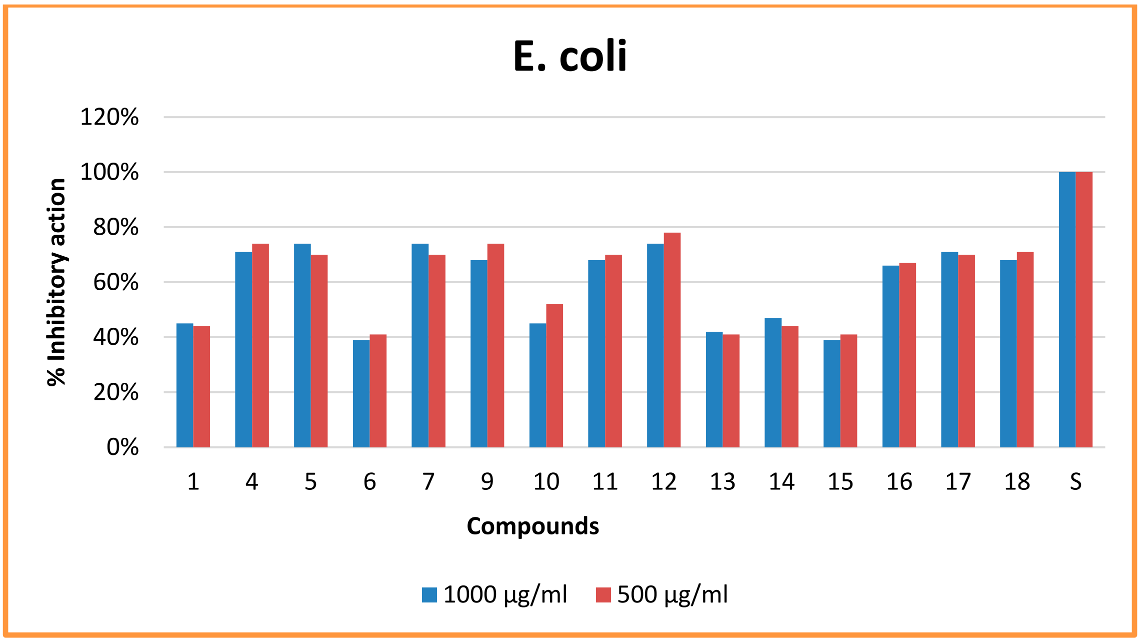


**Chart S3:** The antibacterial efficiency of synthesized compounds against *E. Coli*

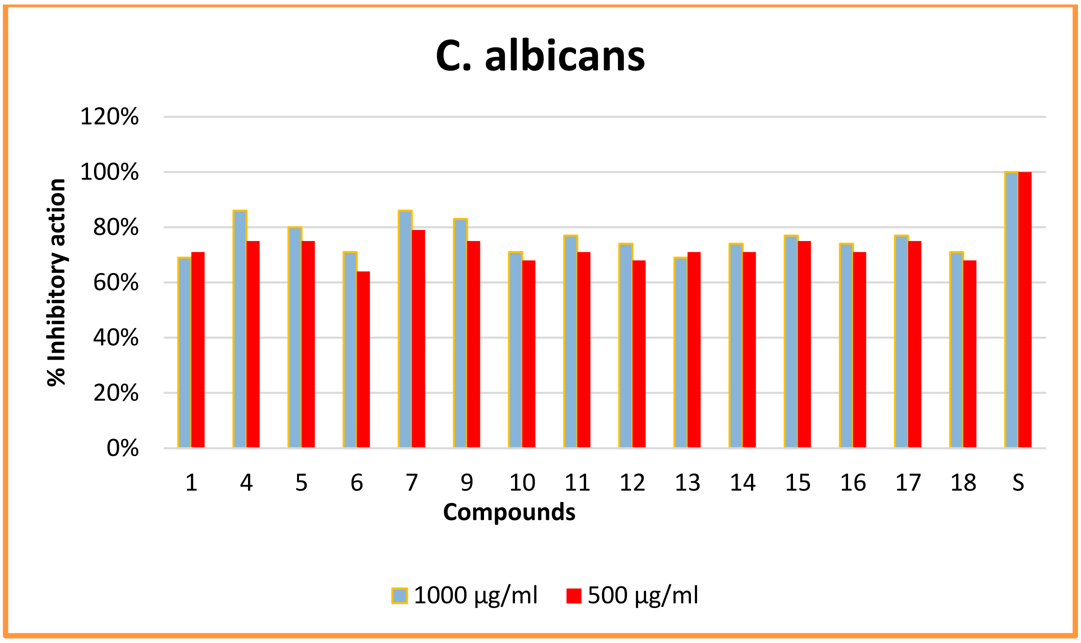


**Chart S4:** The antibacterial efficiency of synthesized compounds against *C. albicans*

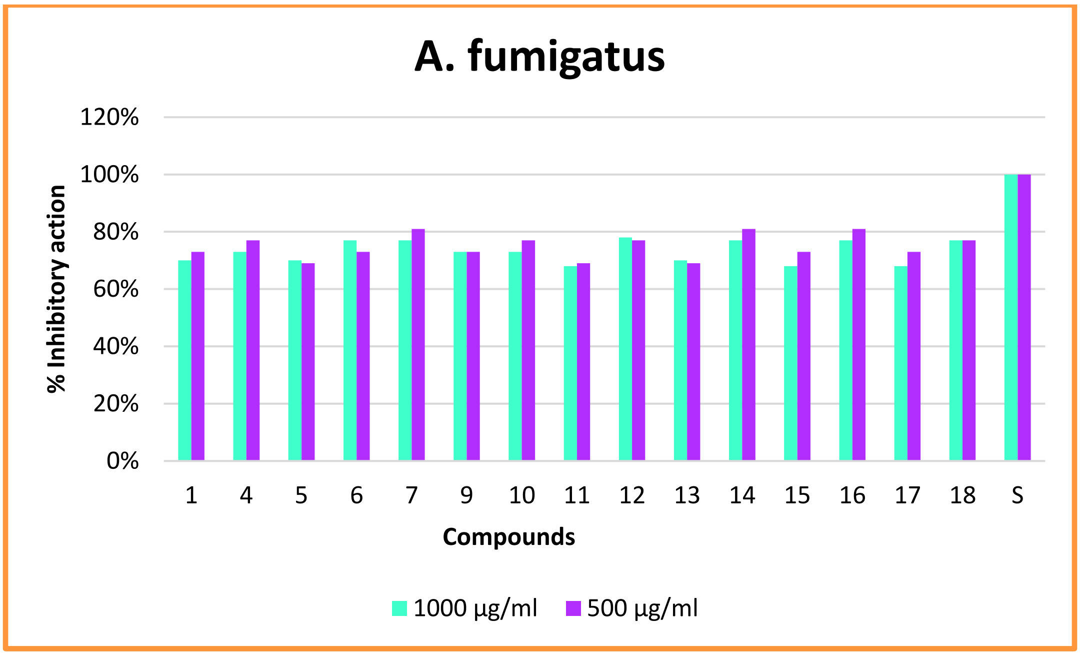


**Chart S5:** The antibacterial efficiency of synthesized compounds against *A. Fumigatus*
